# Developing a Slow-Release Permanganate Composite for Degrading Aquaculture Antibiotics

**DOI:** 10.3390/antibiotics12061025

**Published:** 2023-06-07

**Authors:** Chainarong Sakulthaew, Chanat Chokejaroenrat, Sidaporn Panya, Apisit Songsasen, Kitipong Poomipuen, Saksit Imman, Nopparat Suriyachai, Torpong Kreetachat, Steve Comfort

**Affiliations:** 1 Department of Veterinary Technology, Faculty of Veterinary Technology, Kasetsart University, Bangkok 10900, Thailand; cvtcns@ku.ac.th (C.S.); kitipong.po@ku.th (K.P.); 2 Department of Environmental Technology and Management, Faculty of Environment, Kasetsart University, Bangkok 10900, Thailand; sidaporn.pa@ku.th; 3 Department of Chemistry and Center of Excellence for Innovation in Chemistry, Faculty of Science, Kasetsart University, Bangkok 10900, Thailand; fsciass@ku.ac.th; 4 Integrated Biorefinery Excellent Center (IBC), School of Energy and Environment, University of Phayao, Phayao 56000, Thailand; saksit.im@up.ac.th (S.I.); nopparat.su@up.ac.th (N.S.); torpong.kr@up.ac.th (T.K.); 5 School of Natural Resources, University of Nebraska-Lincoln, Lincoln, NE 68583-0915, USA; scomfort1@unl.edu

**Keywords:** antibiotic removal, binding agents, dispersing agents, permanganate oxidation, release kinetics, slow-release formulations

## Abstract

Copious use of antibiotics in aquaculture farming systems has resulted in surface water contamination in some countries. Our objective was to develop a slow-release oxidant that could be used in situ to reduce antibiotic concentrations in discharges from aquaculture lagoons. We accomplished this by generating a slow-release permanganate (SR-MnO_4_^−^) that was composed of a biodegradable wax and a phosphate-based dispersing agent. Sulfadimethoxine (SDM) and its synergistic antibiotics were used as representative surrogates. Kinetic experiments verified that the antibiotic-MnO_4_^−^ reactions were first-order with respect to MnO_4_^−^ and initial antibiotic concentration (second-order rates: 0.056–0.128 s^−1^ M^−1^). A series of batch experiments showed that solution pH, water matrices, and humic acids impacted SDM degradation efficiency. Degradation plateaus were observed in the presence of humic acids (>20 mgL^−1^), which caused greater MnO_2_ production. A mixture of KMnO_4_/beeswax/paraffin (SRB) at a ratio of 11.5:4:1 (*w*/*w*) was better for biodegradability and the continual release of MnO_4_^−^, but MnO_2_ formation altered release patterns. Adding tetrapotassium pyrophosphate (TKPP) into the composite resulted in delaying MnO_2_ aggregation and increased SDM removal efficiency to 90% due to the increased oxidative sites on the MnO_2_ particle surface. The MnO_4_^−^ release data fit the Siepmann–Peppas model over the long term (t < 48 d) while a Higuchi model provided a better fit for shorter timeframes (t < 8 d). Our flow-through discharge tank system using SRB with TKPP continually reduced the SDM concentration in both DI water and lagoon wastewater. These results support SRB with TKPP as an effective composite for treating antibiotic residues in aquaculture discharge water.

## 1. Introduction

Veterinary antibiotics are indispensable inputs for aquaculture practices. While both prophylactic and therapeutic uses of antibiotics are very effective in promoting aquacultural yields, the subsequent effects of antibiotics on water quality have largely been ignored [1,2,3]. Antibiotic-contaminated discharge water usually receives zero or insufficient treatment prior to being released into downgradient watersheds. Subsequently, these untreated antibiotics may affect the environment by introducing antibiotic-resistant pathogens or killing waterborne microorganisms [4].

In this study, sulfadimethoxine (SDM) was selected as a representative antibiotic because it is the most commonly used sulfonamide antibiotic in veterinary medicine and is administered solely or synergistically with ormetoprim (OMP) and trimethoprim (TMP) [5]. Moreover, previous researchers have documented that SDM-contaminated water can pollute drinking water supplies and may cause environmental threats. For example, Yuan et al. [6] collected samples from natural receiving water and sediment from the Hangzhou Bay area of China and found SDM in the range of 0.59–1.21 ng L^−1^; SDM concentrations in the range 1.73–2.5 ng L^−1^ were detected in drinking water sources for Guilin area, China [7]. Zhou et al. [8] found that SDM was toxic to four aquatic organisms (microalgae, freshwater *Chlorella vulgaris*, marine *Isochrysis galbana*, and *Daphnia magna*). Finally, SDM and other sulfonamide antibiotics are not readily biodegradable; thus, they require a longer time for conventional biological treatment [9]. Therefore, it may be necessary to oxidize the SDM into smaller molecules before applying a biodegradation process.

Removing antibiotics from aquaculture systems presents numerous challenges. Frequently employed technologies, such as chlorination, exhibit limited efficacy and could lead to unexpected ecological consequences from byproduct toxicity [10]. Recently, several techniques have been devised for the removal of antibiotic pollutants from aqueous solutions, including adsorption, photocatalysis, persulfate oxidation, and advanced oxidation processes (AOPs) [11,12,13,14,15]. However, a significant challenge to these techniques mostly pertains to the high levels of dissolved organic carbon concentrations present in the wastewater generated by aquaculture farming. As a result, a large quantity of MnO_4_^−^ is necessary to address this issue. The configurations of the discharge zones in aquaculture lagoons also create chemical application issues, such as how to apply the oxidant and how often.

The efficacy of a slow-release oxidant has been demonstrated in providing a gradual and prolonged release over a period of time, which negates the need for oxidant replenishment. The two most suitable oxidants include persulfate (S_2_O_8_^2−^) and permanganate (MnO_4_^−^). Although slow-release persulfate has shown potential as a remediation option for subsurface contaminants, it typically necessitates an activation method to produce more potent radicals (i.e., SO_4_^·−^) [16,17]. Therefore, the selection of slow-release permanganate (SR-MnO_4_^−^) appears to be more appealing due to its potential for facile implementation [18,19].

Various composites have been developed to produce SR-MnO_4_^−^. The type of binding agent in the formulation, such as paraffin wax, polymer, or cement, is an important factor in MnO_4_^−^ release [19,20,21,22]. Where possible, a biodegradable binding agent material is preferable to a synthetic one [23]. In addition, the manganese dioxide (MnO_2_) that forms during the release of MnO_4_^−^ can block pores used for permanganate diffusion from the SR surface [24]. To date, only a few studies have investigated the releasing mechanisms of SR-MnO_4_^−^ using modeling [25].

Our objective was to develop a slow-release permanganate composite using biowax and a phosphate-based dispersing agent that could be used in situ to reduce antibiotic concentrations in aquaculture lagoons. In this study, we determined changes in the physicochemical properties on the slow-release surfaces, the releasing patterns of permanganate, the optimum composite for maintaining the continual release of permanganate, the influential effects on antibiotic degradation, and the impact environmental conditions had on antibiotic degradation rates.

## 2. Results and Discussion

### 2.1. Antibiotic Kinetic Experiments

Results showed that antibiotic concentrations (SDM, OMP, and TMP) proportionally decreased faster at higher MnO_4_^−^ concentrations or lower initial antibiotic concentrations (Supplementary Materials Figure S1). Quick drops in SDM concentrations were observed, unlike those of OMP and TMP, which displayed a continual decrease (Figure S1A vs. Figure S1B,C). Here, the difference in antibiotic degradation efficiency was solely attributable to where MnO_4_^−^ would tend to attack preferentially, such as the S-N bond of sulfonamide and aniline-SO_2_ [26,27].

Laszakovits et al. [28] reported that MnO_4_^−^ was in excess when the molar ratio of MnO_4_^−^ to contaminant was 5–10, and then the antibiotic destruction rates (k_obs_) can be determined as pseudo 1st order rates (k_obs-SDM_ = 0.017–3.893 h^−1^, k_obs-OMP_ = 0.033–0.514 h^−1^, and k_obs-TMP_ = 0.029–0.307 h^−1^). According to the general rate equation (Equation (1)), the 2nd order rate constant ($k^{n}$) can be calculated from Equations (2) and (3):

(1) $$r=k^{n}{\left[Antib\right]}^{\alpha }{\left[MnO_{4}^{-}\right]}^{\beta }$$

(2) $$r=k_{obs}{\left[Antib\right]}^{\alpha }$$

(3) $$k^{n}=\frac{k_{obs}}{{\left[MnO_{4}^{-}\right]}^{\beta }}$$

where r is the reaction rate, α is the reaction order with respect to antibiotics, and β is the reaction order with respect to MnO_4_^−^.

**Figure 1:**
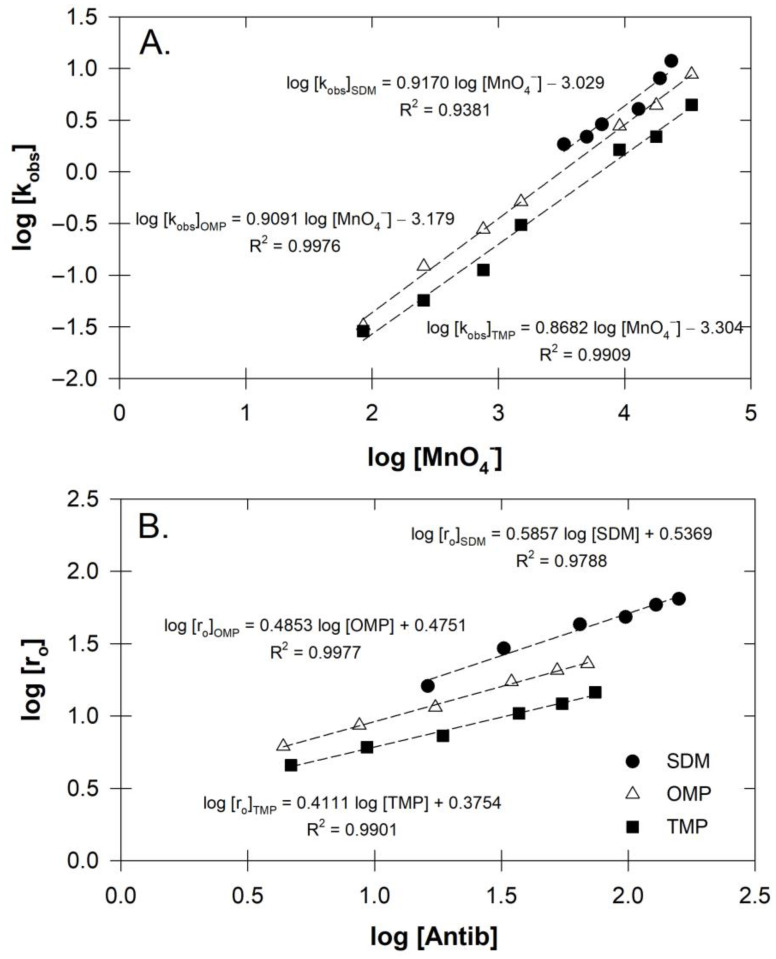
(**A**) Plot of pseudo-order rate constants and various concentrations of MnO_4_^−^ for antibiotics three antibiotics (Sulfadimethoxine, SDM; ormetoprim, OMP; or trimethoprim, TMP) treated with MnO_4_^−^ (**B**) Plot of initial rates and various concentrations of antibiotics when treated with MnO_4_^−^ at 1.133 mM.

By using these equations, these antibiotic-MnO_4_^−^ reactions resulted in second-order rates of 0.128 ± 0.062 s^−1^ M^−1^ for SDM, 0.097 ± 0.005 s^−1^ M^−1^ for OMP, and 0.056 ± 0.008 s^−1^ M^−1^ for TMP (Figure 1). These rates were consistent with the ranges for other antibiotics under similar conditions, such as ciprofloxacin (0.61 s^−1^ M^−1^) [29]. Hassan et al. [30] have suggested that the accelerated degradation rate observed in the presence of MnO_4_^−^ could also be attributed to the presence of other active manganese oxide species (MnO_x_) that may have acted concurrently with MnO_4_^−^, especially at lower solution pH levels. In our case, the organic solvent was not involved in the experimental setup and so could not cause the auto-decomposition of MnO_4_^−^ to produce MnO_2_, as the MnO_4_^−^ concentration ratio was quite high [31]. Therefore, any effect from MnO_x_ during our oxidation process was unlikely.

### 2.2. Effect of Co-Contaminants

The presence of OMP or TMP with SDM resulted in a 3-fold decreased rate of SDM degradation (Figure S2A). Likewise, adding SDM to OMP slowed OMP degradation by 8-fold, and adding SDM to TMP slowed TMP degradation by 6.5-fold (Figure S2B,C). This confirmed our previous results that SDM was preferentially oxidized over OMP and TMP and that the sensitivity of the core molecules to MnO_4_^−^ was the limiting factor for antibiotic degradation. 

Here, the sulfonamide structure was more prone to disruption than the diaminopyridine ring of OMP and TMP. Albeit these three aquaculture antibiotics could be ultimately removed, the required time was quite extended compared to other well-known antibiotics, such as oxytetracycline. The presence of the N atom on the heterocyclic ring of SDM, OMP, and TMP, can minimize the electron density on the rings and deflect the attack by MnO_4_^−^ to initiate the ring cleavage [32].

### 2.3. Effect of Initial pH

**Figure 2:**
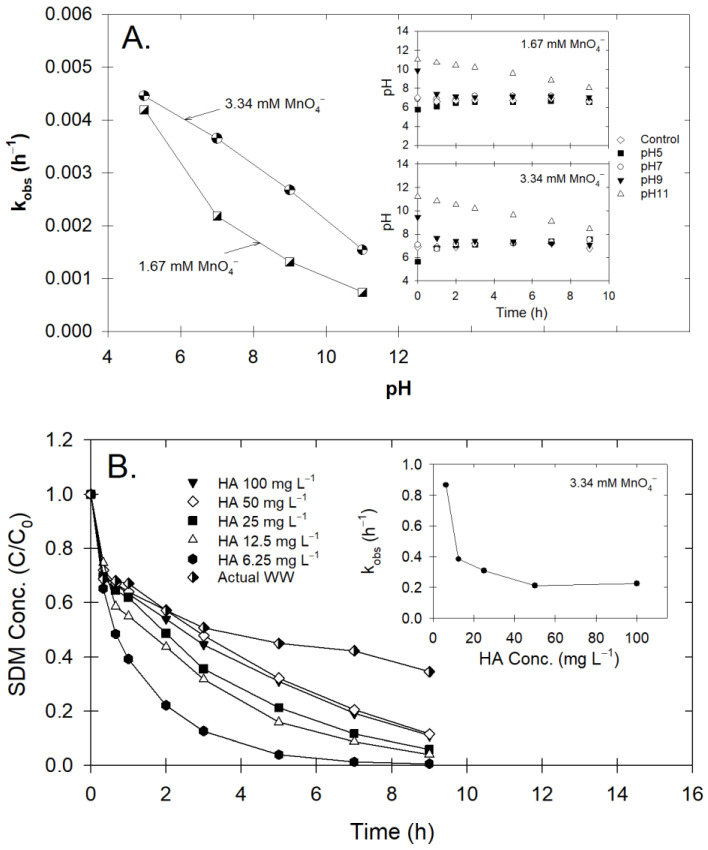
(**A**) Observed kinetic rate constant (k_obs_) of SDM degradation with different initial pH levels following treatment with MnO_4_^−^. Inset graph shows temporal changes of pH of corresponding MnO_4_^−^ concentration. (**B**) Temporal changes of SDM concentration following treatment with MnO_4_^−^ under varying humic acid concentrations or actual wastewater discharge. Inset graph shows comparison of k_obs_ at corresponding HA concentration.

We observed changes from the initial pH level toward a neutral pH and a slight decrease of MnO_4_^−^ (inset of Figure 2A). Using twice as much MnO_4_^−^ (3.34 mM) also produced similar changes in MnO_4_^−^ and pH. As the MnO_4_^−^ reaction proceeds, the Mn–byproduct (MnO_2_) will naturally form, and the pH will more likely be in the range of 4–6. This would allow the MnO_2_ to enhance the oxidative performance, resulting in a faster reaction in this pH range [27]. Although MnO_2_ can catalyze oxidative reactions, it could negatively impact our slow-release MnO_4_^−^. MnO_2_ can also block MnO_4_^−^ releasing passage from the slow-release composite, which would delay contaminant degradation. Therefore, minimizing MnO_2_ during treatment was an important research niche for developing a slow-release oxidant composite for aquacultural systems.

### 2.4. Effect of Humic Acids and Real Wastewater

Results showed that the k_obs_ decreased with increasing humic acid, indicating the strong influence of HM on SDM degradation (Figure 2B). Conversely, Sun et al. [33] reported that the presence of HM increased contaminant removal efficiency via the formation of a secondary oxidant (MnO_2_) during the MnO_4_^−^ reaction. However, the increased k_obs_ did not appear in our experiments, perhaps due to several reasons: (1) the operating pH (unbuffered pH) did not facilitate MnO_2_ formation; (2) the SDM-MnO_4_^−^ rate was quite slow compared to the tentative reaction time of MnO_2_ with other contaminants, which usually occurred within the first 30 min; and (3) over time, the MnO_4_^−^ concentration was unchanged, indicating that if MnO_2_ did form, it might be insufficient to initiate MnO_2_ oxidation. Notably, k_obs_ values were unchanged at high HM concentrations; in addition, the SDM relative concentration seemed to reach a plateau sooner with the wastewater compared to the 100 mg L^−1^ HM solution (Figure 2B). Here, MnO_2_ may have been readily liberated as the MnO_4_^−^ was surrounded by organic constituents that are prone to react with any oxidative substance. 

Given these possibilities, previous reports also demonstrated that the interaction of organic matter with oxidative molecules was quite complex; thus, different types of impact may be expected depending on the oxidant. For example, phenolic moieties in organic matter may also act as an activator for persulfate oxidation, which would result in a much faster degradation rate [34]. However, our results showed that humic substances could have a major inhibitory effect on SDM degradation, delaying it by as much as 50% compared to the control (no HM; Figure S1 vs. Figure 2B). Similar observations showed that, at only 5 mg L^−1^ of HM, the degradation of sulfamethoxazole was inhibited during MnO_4_^−^ oxidation (Gao et al., 2014). Therefore, prolonging the contact time of the oxidant and having a slightly higher MnO_4_^−^ concentration must be considered for real-world applications. The aforementioned statements provide sufficient proof to support the beneficial application of slow-release MnO_4_^−^.

### 2.5. Release Concentration of SR Permanganate

#### 2.5.1. Release Concentration

Using paraffin and no biowax, a rigid cylindrical shape was produced that provided the continual release of MnO_4_^−^ up to ~500 mg L^−1^, which was nearly 95% of MnO_4_^−^ in one SR (Figure S3B). Because paraffin mostly contains saturated long-chain hydrocarbons (C18–C60), its biodegradation can take some time. Furthermore, Carrilloa et al. [35] reported that the accumulation of paraffin wax can cause severe health effects on aquatic life and their habitat, which could also threaten human health.

During the preparation of slow-release samples, we found that the soy wax-paraffin-MnO_4_^−^ mixture was unlikely to form. The mixture’s homogeneity was so sparse that the material was crumbly with an obvious covering of wax. These crumbs provided individual encapsulation that would have served as many SR-MnO_4_^−^ sites and therefore provided higher MnO_4_^−^ release (Figure S3C). The deformation of soy wax may have been due to its being more branched with short-chain fatty acids, hydroxyl groups, and containing more ester compounds, making it very difficult to form a rigid SR [23,36]. In addition, soy wax thermographs from differential scanning calorimetry support its ability to melt at a lower temperature compared to paraffin and beeswax [37]. We believe that these abilities may cause deformity of the mixture and its failure to re-solidify into the desired shape at room temperature, resulting in undesirable shredding. However, our current results showed that the releasing concentration was quite low (<350 mg L^−1^) as most KMnO_4_ granules were entirely covered with unmixed waxes that minimized the surface diffusion channel, worsening the release of MnO_4_^−^ (Figure S3C). 

As discussed earlier, the physicochemical properties of waxes play an important role in the releasing ability of MnO_4_^−^. The rice bran wax chemical composition was ester compounds (up to 73.4%), triacylglycerols (21.9%), and free aliphatic alcohol (4.6%) [38]. Here, rice bran wax failed to form a rigid shape with any of the mixtures as the MnO_4_^−^ releasing concentrations were inconsistent for both short-term (<7 d) and long term (>7 d) release, resulting in large variations in the MnO_4_^−^ concentration (Figure S3D). In addition, the rice bran wax tended to swell in water in our separate swelling test experiment. Therefore, rice bran wax was not suitable as a binding agent for SR-MnO_4_^−^.

On the other hand, beeswax performed very similarly to using paraffin alone, despite lessening the amount of paraffin in the wax proportion, resulting in a spongier surface. In terms of releasing MnO_4_^−^ concentration, large concentration discrepancies were observed between samples from 0.25 d to 7 d and from 28 d to 56 d. In contrast, the releasing concentration was quite consistent from 7 d to 28 d (Figure S3E). Compared to the paraffin, the initial phase of beeswax provided 1-fold more releasing concentration, indicating that beeswax was a better binding agent than paraffin alone (Figure S3B vs. Figure S3E). With time, oxidation of MnO_4_^−^ on the beeswax slowly occurred, with the possible formation of MnO_2_, resulting in blockage of the diffusing channel of MnO_4_^−^ during 7 d to 28 d. We observed more obvious cracks on the SR surface on day 28, which created new diffusing channels, resulting in more MnO_4_^−^ concentration being released. At 56 d, the highest concentrations of beeswax at all ratios were still 17% lower than from using paraffin alone (Figure S3E).

In terms of chemical composition, beeswax consists of longer chain carbons compared to soy wax. One of the major components of beeswax is esters, which contain up to 52 carbons and a series fraction of internal chain methylene (int-(CH_2_)) [39]. Therefore, the beeswax can degrade more easily than paraffin.

#### 2.5.2. SR-MnO_4_^−^ Surface Properties

**Figure 3:**
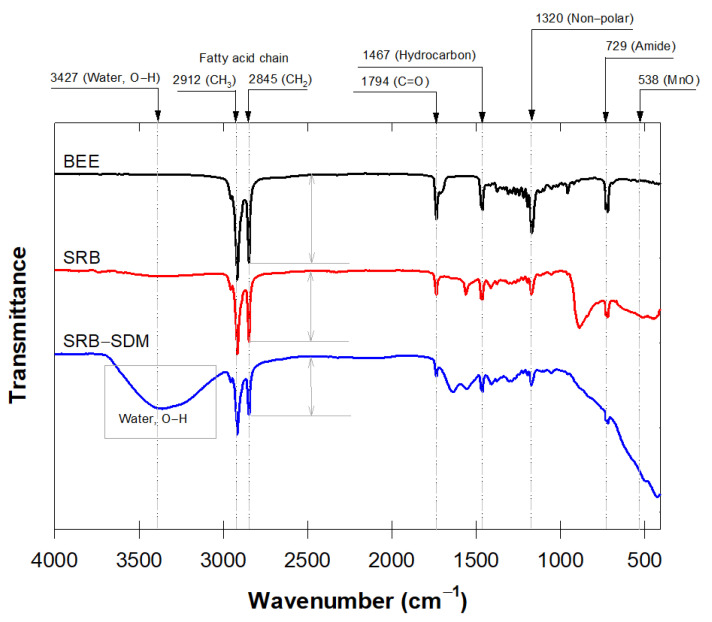
Fourier-transform infrared spectroscopy spectra of three different SRs: pure beeswax (BEE), slow-release permanganate consisted beeswax in the mixture (SRB), and SRB after soaked in SDM solution.

We initially selected beeswax SR-MnO_4_^−^ (SRB) to further characterize its changes in surface properties using FTIR (Figure 3). The double peaks at 2912–2845 cm^−1^ were attributed to the presence of fatty acid chains, while peaks at 1320 and 729 cm^−1^ were ascribed to C-H stretching in symmetry with aliphatic hydrocarbons and the amide group. These spectra resembled the major component of natural beeswax [40]. The signals at 1467 and 1794 cm^−1^ belonged to the C=C stretching band of saturated hydrocarbons and the C=O stretching vibration in the wax polymer.

The peak intensities decreased with a decreased portion of beeswax in the SRB and SRB-SDM, while still showing the original components of beeswax (Figure 3). This decrease may imply that the short longevity of SRB improved its suitability for being biodegradable. The absence of the CH_2_ rocking bands at 800 cm^−1^ on the SRB-SDM indicated the loss of the crystal structure of the hydrocarbon chain due to MnO_4_^−^ oxidation on the SR surface during the batch experiment. Shaabani et al. [41] reported that MnO_4_^−^ oxidation was responsible for shortening the aliphatic hydrocarbon chain, causing the disappearance of the FTIR bands. The 538 cm^−1^ band corresponded to the stretching vibration of the adsorption band of MnO on the MnO_2_ molecular structure that resulted from the SDM-MnO_4_^−^ reaction [42]. Therefore, it could be concluded that the MnO_2_ rind that appeared on the SRB surface during oxidation could later block the MnO_4_^−^ diffusing passage. This meant that chemical additions, such as dispersing agents, were needed to prevent the aggregation of MnO_2_ and simultaneously enlarge the release passage.

#### 2.5.3. Chemical Addition/MnO_4_^−^ Residual on Surface

In this releasing experiment of mixture set B, we selectively presented the controls (XC0) and ones denoted as XT1, XT2, XS1, and XS2 where X represents the type of wax—S, R, B, or P—in the successive releasing experiment (Figure S4). Despite adding the TKPP or SHMP to benefit the emulsifying activity and support gel formation in the mixture [43], at higher amounts (>0.04 g), our SR failed to achieve the desired cylindrical shape after one week (Table S4). This is because increasing the dispersing agent by more than 2.5% of the total SR weight—the total of binding agents was then less than 24.7%—could easily dissolve in water and leave voids in the SR surface, making it unstable to maintain the original shape (Table S4).

We found that in the short term, chemical addition made only minor differences in the MnO_4_^−^ release compared to previous experiments with no chemical addition (Figure S3 vs. Figure S4). Soy wax and rice bran wax still presented oscillated concentrations due to unsuitability between the binding agent and MnO_4_^−^, while beeswax and paraffin provided more stable release. Among these various tests, BT2 and BS2 provided the best releasing concentration (Figure S4), which was approximately 20% better than without chemical addition (Figure S3). This was due to the phosphate ions binding with the colloidal manganese oxide, resulting in the creation of repulsive forces that later delayed MnO_2_ aggregation.

#### 2.5.4. Releasing Empirical Formula

Generally, release MnO_4_^−^ concentration showed fresh dissolution in the initial phase, followed by continual release until reaching the saturation plateau (Figure S6). Although most of the MnO_4_^−^ release patterns had similar trends, the release kinetics differed depending on various types of mixtures and amounts of binding agent (Table 1). The MnO_4_^−^ release pattern can be varied depending on the uniformity of the mixture and the granule-aligning configuration of the SR. Biphasic graph types of the release kinetics were observed in all the SR formulations, confirming the common pattern for oxidant release, as our research group previously demonstrated (Table 1; Figures S6–S10). We evaluated a full range of experimental times (60 d) for all the theoretical models, except the Higuchi models, in which the partial time (~60% of released concentration; ~8 d) was separately evaluated, as suggested by Passot et al. [44]. The results indicated that beeswax and paraffin had longer steady state time spans (11 d vs. 15 d) than those of soy wax and rice bran wax (~5 d; Figure S7). Therefore, linear regression for the shorter timespan (<8 d) for the Higuchi model provided a better fit (Figure S8).

All of the r^2^_adj_ values obtained using the Noyes-Whitney model were unsatisfactory due to the slight increase toward the end of releasing experiments and its possessing biphasic behavior (Table 1; Figure S9). The Noyes-Whitney model calculation is based on a uniform layer, while our SR was manufactured from a mixture of binding agents, which may not have uniformly encapsulated both granules of MnO_4_^−^ and the dispersing agents. In addition, we observed that only beeswax with dispersing agents (BT1, BT2, and BS1) could provide a better fit within the first 15 d of the experiment (r^2^_adj_ > 0.87). This might have been due to the texture of the beeswax itself, which allowed for more uniform mixing from the circumferential surface toward the center of the SR cylinder. In addition, TKPP and biowax were better distributed in the SR mixture than SHMP. Unlike the Higuchi model, it was clear that the Noyes-Whitney model would only be suitable for slow-release types that had reached 80% of the released concentration.

The lack of correlation using the Weibull model was observed for soy wax and rice bran wax (r^2^_adj_ values of 0.73–0.84; Table 1; Figure S10). Because of the obvious biphasic feature of the release pattern in these two types of biowax, it was unlikely to achieve a well-fitted pattern with a Weibull model. Unlike the beeswax and paraffin SR, the r^2^_adj_ was better described with the Weibull model. Furthermore, the shape parameters (β values) of 0.3667–0.5269 in all formulations implied that the SR released MnO_4_^−^ according to Fickian diffusion [45].

Among the other models, the Higuchi model could better provide phenomenological analysis of releasing data, but only within the recommended timeline [44]. None of the r^2^_adj_ values for SR manufacturing with soy wax and rice bran wax were acceptable in the full timespan range (Table 1), confirming that these SR types did not correlate well with this model using the entire timespan and that these waxes contributed to the random release of MnO_4_^−^, even in the initial phase. These physical wax characteristics were so inconsistent that the wax texture prevented the mixture uniformity. The uneven mixture was probably the main reason causing the rind and wax blockage on the MnO_4_^−^ dissolution front.

Considering only t < 8 d, the r^2^_adj_ values using soy wax and rice bran wax were still unsatisfactory, with the paraffin and beeswax applications providing much better fits (r^2^_adj_ 0.944–0.991; Table 1). In addition, when paraffin was used with TKPP or SHMP addition, the k values were more consistent compared to those using beeswax, indicating that paraffin could provide a likely controllable release (Table 1). The beeswax was more likely controllable with TKPP addition than SHMP addition.

Overall, in the beeswax formulations, TKPP addition produced a better fit and slightly lower k values than SHMP addition. By extending to the full range of release analysis, the Siepmann-Peppas model, based on the power law model, was better suited with much higher r^2^_adj_ values and could better predict the release of MnO_4_^−^ from the SR mixture formulation. Similar to the Higuchi model, only formulations with beeswax or paraffin only provided relatively high r^2^_adj_ values > 0.9. The only exception was the SHMP addition in the beeswax formulation that provided a relatively low r^2^_adj_ value, indicating that SHMP might not be a good candidate to provide constant MnO_4_^−^ release.

The results obtained by applying the Siepmann-Peppas model showed that this model was most suitable for full-range analysis of the release of MnO_4_^−^. The release longevity revealed that the MnO_4_^−^ reached its maximum capacity no later than 20 d (Figure S6). It also revealed that the MnO_4_^−^ releasing trends were deliberate when the α and β values were lower than 200 and relatively close to 0.300 (Table 1). In other words, a high amount of chemical addition would either produce an out-of-shape SR cylinder or make the release pattern unpredictably random. 

In addition, from a structural wax standpoint, both paraffin and beeswax contain up to 90% CH_2_ carbons, but beeswax also contains larger amounts of polar compounds, such as alcohols, free acids, and esters, [46]. When SRB meets water, part of the beeswax would swell and possibly hinder the release of MnO_4_^−^ by partially blocking the diffusion channel. This would be unlikely to occur with paraffin as it contains mostly alkane groups, which are hydrophobic. Therefore, there could have been several pores on the SR surface once MnO_4_^−^ started to diffuse, making it easier to control chemical release.

#### 2.5.5. Comparison of SDM Degradations by MnO_4_^−^ Solution and SR-MnO_4_^−^

**Figure 4:**
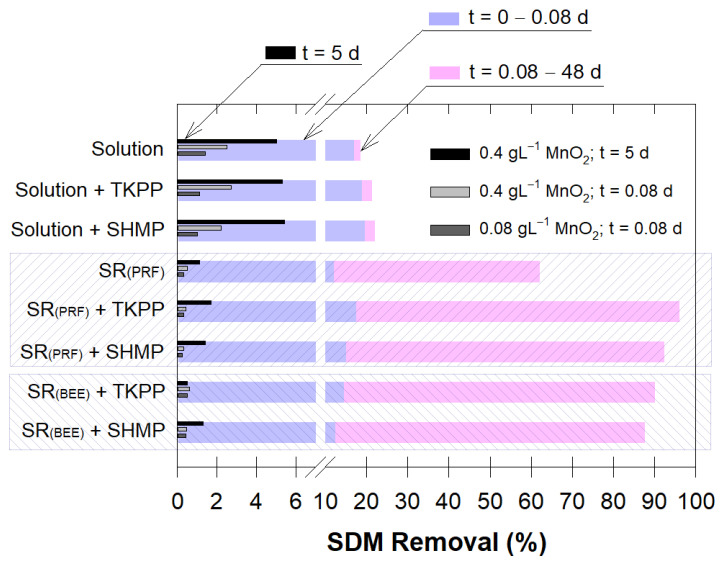
SDM removal percentage with different treatments of permanganate (solution or SR) for short-term (0.08 d) and long-term (48 d). Embedded bar graphs represent SDM removal percentages for different treatments of MnO_2_ at varying MnO_2_ amounts. The MnO_2_ treatment used a similar configuration (solution or SR) to that of the corresponding MnO_4_^−^ bar graph.

Results showed that the MnO_4_^−^ solution alone removed SDM better than the composites in the short term (~0.08 d), while the SR composites performed much better over the long term (up to 48 d) (Figure 4). A dispersing agent in SR-MnO_4_^−^ revealed up to 20–30% better SDM removal efficiency (Figure 4). This indicated that both TKPP and SHMP could perfectly delay MnO_2_ aggregation.

The oxidation of MnO_2_ alone with SDM showed that the SDM removal was proportional to the amount of MnO_2_, but to a lesser extent than for MnO_4_^−^ and that the presence of chemical addition did not change the SDM removal efficiency (see embedded bars in Figure 4). In addition, no adsorption of SDM on the MnO_2_ surface was observed; rather, it has been shown to easily degrade with the initiation of electron transfer (Gao et al., 2012). Furthermore, the available oxidative sites on the MnO_2_ surface could be hindered by binding agent embedment on the MnO_2_ particles. The formation of rind on the SR surface could be minimized by using a dispersing agent, which allowed more MnO_4_^−^ to be released into the solution. 

The paraffin SR-MnO_4_^−^ was better at releasing MnO_4_^−^ and degrading SDM compared to the beeswax SR-MnO_4_^−^ (Figure 4, Figures S3 and S4). However, adding TKPP was a better combination with beeswax than adding SHMP, regardless of these two SR types. This was due to the smaller phosphate group attached to the TKPP molecules (diphosphate or pyrophosphate) (Table S2) that allowed better chelating ability on metal ions and the shorter chained polyphosphates of TKPP, giving it approximately two-fold greater water solubility than SHMP [47].

To ensure the absence of MnO_2_ on the SR surface of SRB with TKPP addition, we proved the MnO_2_ formation using XRD. By comparing the results of four different types of SRB with the various polymorphs of the MnO_2_ standard (pyrolusite, ramsdellite, and hollandite), there was no matching of any MnO_2_ formation on the surface, indicating that TKPP could successfully prevent self-aggregation of MnO_2_ (Figure S5). Notably, there was also no clear evidence of colloidal or other precipitates in the solution.

### 2.6. SR Permanganate Use in Contact Tank

**Figure 5:**
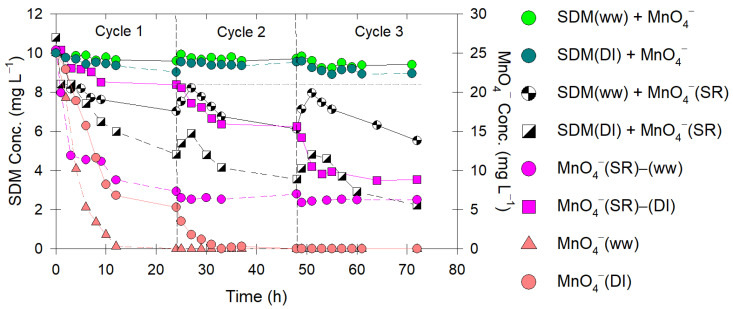
Temporal changes in SDM concentration (C/C_o_) and MnO_4_^−^ concentration observed in contact tank following treatment with different water matrices (DI water or actual wastewater).

Results showed that the MnO_4_^−^ solution could not decrease the SDM concentration in both matrices and may even have slightly increased the overall SDM concentration with time because of the continuous flow of newly flushed SDM-contaminated water into the system (Figure 5). This indicated a possible adverse effect when KMnO_4_ was selected as the sole treatment. Although there was a slight decrease in the SDM concentration in the first cycle, the available MnO_4_^−^ in the contact tank may have been insufficient for successive flushing cycles.

When SRB (i.e., with TKPP) was used, the results showed that both the SDM concentration and the MnO_4_^−^ residual concentration continually decreased with time (Figure 5). Again, a slight increase in the SDM concentration was observed. The MnO_4_^−^ concentration was less than the total concentration in the releasing experiment because of the sizing difference. However, with this low concentration of MnO_4_^−^ prior to entering the effluent reservoir, we suggest that numerous SRB types could be used and the contact time could be extended to facilitate system efficiency.

Because we expected that other organic contents would affect our system and our SRB, we mimicked the previous experiment with the actual aquaculture discharge water (ww). Similar decreasing trends in both the SDM and MnO_4_^−^ concentrations were observed. However, after the first cycle, the overall removal percentage of SDM reached ~27% and ~55% for DI water and actual wastewater and continued to decrease with time (Figure 5). The addition of TKPP proved to prolong the slow release of MnO_4_^−^, delay the formation of MnO_2_, and negate the need for frequent replenishment of the SRB.

Based on the SDM removal efficiency and the MnO_4_^−^ concentration, the contact tank experiment showed less SDM removal and a lower MnO_4_^−^ concentration than the batch experiment (Figure 5 vs. Figure S1). This could have been due to differences in the contact tank volume, indicating that regular cleaning practices and environmental conditions on the farm may need to be further evaluated with our developed SR to efficiently remove these contaminants from farming wastewater. Overall, we proved that SRB+TKPP was more effective than using MnO_4_^−^ solution alone, provided that the existence of organic constituents in the wastewater was taken into consideration.

## 3. Materials and Methods

### 3.1. Chemicals and Analyses

The chemicals used in experiments were purchased from several vendors. Sulfadimethoxine (C_12_H_14_N_4_O_4_S: 122-11-2, SDM), ormetoprim (C_14_H_18_N_4_O_2_: 6981-18-6, OMP), and trimethoprim (C_14_H_18_N_4_O_3_: 738-70-5, TMP; Table S1) were obtained from Dr. Ehrenstorfer GmbH (Wesel, Germany). Manganese dioxide (MnO_2_) was purchased from BDH (Poole, England). Potassium permanganate (KMnO_4_), ascorbic acid, tetrapotassium pyrophosphate (TKPP), and sodium Hexametaphosphate (SHMP; Table S2) were of analytical reagent (AR) grade and purchased from Ajax Finechem (Oakland, New Zealand). Humic acid was obtained from Sigma-Aldrich (St. Louis, MO, USA). All SR binding agents (synthetic paraffin, paraffin, soy wax, beeswax, and rice bran wax) were acquired from Chemipan (Bangkok, Thailand), a local wax manufacturing company in Bangkok, Thailand.

Changes in antibiotic concentration were determined based on high-performance liquid chromatography (HPLC) with an e2695 unit using a diode-array UV detector no. 2998 (Waters, Milford, MA, USA). For the isocratic elution of acetonitrile, 0.1% acetic acid (60:40) was used as the mobile phase at a flow rate of 1 mL min^−1^. The detection wavelength was set at 270 nm for SDM analysis and at 200 nm for OMP or TMP analysis. After injecting 20 µL of samples, antibiotics were separated using a Mightysil RP-18GP column (250 × ∅ 4.6 mm, 5 µm) coupled with a guard column. The MnO_4_^−^ concentration was measured using a Cary60 Agilent UV-Vis spectrophotometer (Santa Clara, CA, USA) at a wavelength of 525 nm.

The SR samples made of selected binding agents were selectively analyzed for surface properties. Fourier-transform infrared spectroscopy (FTIR; Bruker Tensor 27; Billerica, MA, USA) was used to analyze the surface functional groups of the unheated beeswax (BEE), SR-MnO_4_^−^ made of beeswax (SRB), and 7 d SDM-soaked solution SRB (SRB-SDM). To further confirm the absence of MnO_2_ following adding chemical addition (TKPP), a 2θ scan (15–80°) was performed using X-ray diffraction (XRD; Bruker D2 Phaser; Billerica, MA, USA). Comparisons were made after using the SRB with and without TKPP on testing with SDM by soaking in SDM solution for 7 d.

### 3.2. Antibiotic Kinetic Experiments

The first experiment was to determine the MnO_4_^−^ degradation efficiency of antibiotics in a series of batch experiments. A 250 mL Erlenmeyer flask was used as an experimental unit for a 100 mL aqueous solution. Unless stated otherwise, all experiment units were covered with aluminum foil to prevent photodegradation of MnO_4_^−^. All experiments were performed under agitation using an orbital shaker at 150 rpm. Treated samples were quenched to further prevent antibiotic transformation following treatment with MnO_4_^−^. We used ascorbic acid as a quenching agent instead of using manganese salts to avoid interference with the properties of aliquot samples. The typical quenching procedure involved transferring a 1 mL sample at preselected times into a 1.5 mL centrifuge tube that contained 0.1 mL of freshly prepared ascorbic acid (20,000 mg L^−1^), centrifuging at 14,000 rpm for 10 min, removing the supernatant to an HPLC vial, and storing samples until analysis based on HPLC.

Although SDM was the main focus of this research, OMP and TMP were also selected for antibiotic kinetic experiments as they act synergistically with SDM at a 5:1 ratio in real-world medicinal applications [5]. To determine antibiotic reaction rates, we performed batch experiments where the SDM initial concentration was fixed at 161.12 μM and the MnO_4_^−^ concentrations ranged from 0.315 to 5.033 mM. Based on the applicable ratio, OMP or TMP was fixed at 36.45 μM and 34.44 μM, and the MnO_4_^−^ concentrations ranged from 0.189 to 27.181 mM. These high concentration ranges of MnO_4_^−^ allowed us to evaluate the reaction rates when the MnO_4_^−^ was in excess. 

Likewise, using the initial MnO_4_^−^ concentration at 1.133 mM, we treated varying concentrations of either SDM (16.11 to 161.12 μM) or OMP (4.56 to 72.91 μM) or TMP (4.56 to 72.91 μM) individually. The initial rate method modified from Sakulthaew and Chokejaroenrat [19] was selected to determine the kinetic order rates of the antibiotic and MnO_4_^−^.

In addition, we conducted a series of experiments that compared degradation rates when antibiotics were treated alone and in combination (i.e., as co-contaminants) to quantify SDM degradation in the presence of other synergistic antibiotics (OMP and TMP).

### 3.3. Influential Effects on Antibiotic Degradation

#### 3.3.1. Effect of pH

The ambient pH of aquaculture water can fluctuate due to the excreted ammonia from fish following protein feeds, which can cause a slightly higher pH in the discharge water [48]. Therefore, it would be more difficult to degrade antibiotics because MnO_4_^−^ is more efficient in acidic solutions. Therefore, we conducted a series of batch experiments to verify that the SDM destruction rates by MnO_4_^−^ (1.67 and 3.32 mM) were similar at differing levels of the initial pH. The experiment was investigated over a pH range of 3–11 to cover a vital range (4–11). The solution pH was adjusted to the designated pH using either 0.1 M NaOH or 0.1 M HCl. A stock solution of SDM was spiked into the solution to obtain the final concentration of 161.12 μM. Samples were collected periodically following the monitoring of SDM concentrations and pH measurement of the solution.

#### 3.3.2. Effect of Humic Acids and Real Wastewater

Aside from the micropollutant contamination in the discharge water from aquaculture farming, high levels of organic constituents can be a major contributing factor in scavenging for available MnO_4_^−^. In separate sets of vessels, we used a 3.32 mM solution of MnO_4_^−^ and varied the humic acid concentration ranging from 6.25 to 100 mg L^−1^, which was used as a representative of natural organic matter (NOM). To test the treatability of MnO_4_^−^ on-site treatment for SDM, we used real discharge water as the solution matrix in the batch experiment. This wastewater was provided from local prawn farms in Kampangsaen district, Nakhon Pathom province, Thailand, and was collected during the harvesting period. Its water characteristics are presented in Table S3. Similar to most aquaculture farming in rural areas, this water had received insufficient treatment prior to disposal in the adjacent lagoon watershed.

### 3.4. Slow-Release Permanganate

A series of ratios between solid wax (acting as a binding agent), KMnO_4_, and stabilization aids are discussed later in this section. The mixture was heated until the liquid was on a hotplate at 75 °C and continuously stirred to achieve textural homogeneity prior to pouring it into a cylindrical mold (∅ 0.6 cm). Each SR sample was trimmed and weighed to 0.75 ± 0.05 g to ensure minimal fluctuation in the release of the MnO_4_^−^ concentration. These samples were kept in a desiccator at room temperature prior to use within 5 d.

#### 3.4.1. Manufacturing Mixture Ratio

To determine the most optimal mixture for SR-MnO_4_^−^, two types of mixture ratios (A and B) were used for different purposes. The set A mixture was used to evaluate the best slow-but-sustained release of MnO_4_^−^ using different kinds of binding agents (i.e., synthetic paraffin and three types of biowax), whereas the set B mixture was used to evaluate the most suitable type and the amount of dispersing agent (TKPP or SHMP) in the SR mixture. Other research proved that SHMP facilitated a more consistent release of MnO_4_^−^ [24], and both SHMP and TKPP allowed MnO_4_^−^ to enter low permeable zones much deeper than without using these chemical agents in the transport experiments [49].

For mixture set A, we used different amounts of each composition while maintaining the same total weight (3.3 g per batch). In general, biowax was more difficult to solidify compared to paraffin. In each batch, we varied the amount of biowax (0.2–1.0 g per batch) and the paraffin (intervals of 20% or 0–0.8 g per batch) while maintaining the same amount of KMnO_4_ (Figure S3A). We selected this mixture ratio for the succeeding experiments based on two criteria: (1) the best slow-but-sustained release of MnO_4_^−^ and (2) the ability of SR-MnO_4_^−^ to retain its cylindrical shape.

Mixture set B involved the addition of stabilization aids or dispersing agents. The TKPP and SHMP were reported to reduce MnO_2_ rind formation substantially during the sweeping of MnO_4_^−^ flushing in the subsurface [49]. The new mixtures were investigated using varying amounts of the stabilization aid (0.01, 0.02, 0.04, or 0.08 g) (Table S4). To elucidate the effect of the dispersing agents, we maintained the weight of each SR-MnO_4_^−^ at 0.75 ± 0.05 g and the constant amount of paraffin at 0.09 g. Therefore, we compensated for the addition of either TKPP or SHMP by reducing the amount of biowax (Table S4). Here, each type of SR is abbreviated and written as wax·chemical additive·additive amount (Figure S4; Table S4). For example, ST2 was used as the abbreviation for soy wax mixed with 0.02 g of TKPP (per SR). The additional criterion for selecting the most suitable proportion was that the SR should be able to continually release MnO_4_^−^ in the aqueous solution while preventing rind formation.

#### 3.4.2. MnO_4_^−^ Releasing Experiments

Batch experiments were conducted for MnO_4_^−^ releasing to evaluate the optimum ratio for each mixture set. Typical experiments involved placing the SR-MnO_4_^−^ into individual 1.5 L flasks that contained 1 L of water. Each container was thoroughly covered with aluminum foil to prevent MnO_4_^−^ photodegradation. The SR-MnO_4_^−^ was suspended 10 cm from the top of the water surface using cheesecloth bags that allowed 100% MnO_4_^−^ diffusion. Each unit was done in quadruplicate. Sampling for MnO_4_^−^ concentrations occurred immediately after the SR-MnO_4_^−^ had been removed from the flasks. The deformity of SR was recorded as not applicable and was not considered in the succeeding experiments.

#### 3.4.3. Slow-Release Applicability Test

By flushing SR with fresh water, the MnO_4_^−^ concentration is quickly released via diffusion mechanisms because rind formation and different binding agents could interfere with the release of MnO_4_^−^. In an individual container, each SR sample from mixture set B was soaked in 1 L of water for 2 h to imitate the flushing event on aquaculture farms. The MnO_4_^−^ concentration was monitored 12 times during 28 d. Then, we removed the SR from the soaking unit and left it at room temperature until the next sampling time. Four empirical formulas (Siepmann-Peppas, Higuchi, Noyes-Whitney, and Weibull) were selected to quantify the releasing mechanism and better understand the MnO_4_^−^ release. Both the coefficient of determination (R^2^), obtained using the graphical software, and the adjustable coefficient (r^2^_adj_) (Equation (4)) [50] were applied to evaluate the best fitting model for MnO_4_^−^ release.

(4) $$r_{adj}^{2}=\frac{\left(1-R^{2}\right)\left(N-1\right)}{\left(N-m-1\right)}$$

where N is the number of samples in each run and m is the number of parameters in each empirical model. As one of the criteria of SR selection was to maintain its cylindrical shape, we only selected ones with sufficient binding agent (dispersing agent < 2.5%) for further discussion on the releasing model.

To compare the SDM removal using MnO_4_^−^ solution and SR-MnO_4_^−^, we conducted additional experiments that monitored SDM concentrations up to 48 d. Either TKPP or SHMP was included in both treatments (i.e., MnO_4_^−^ solution and SR-MnO_4_^−^). For SR-MnO_4_^−^, we compared only two types of binding agents: (1) paraffin and (2) beeswax. To further evaluate if MnO_2_ could serve as an oxidative surface for SDM in our treatment configuration, we added either 0.08 g L^−1^ or 0.4 g L^−1^ MnO_2_ to the experimental unit. 

### 3.5. Contact Tank Experiment

**Figure 6:**
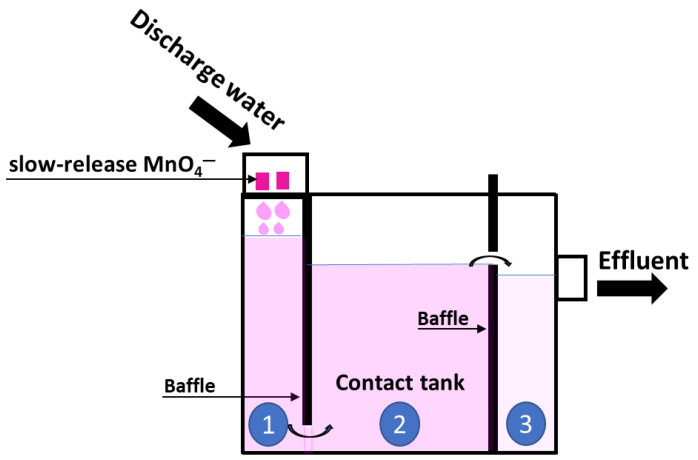
Contact tank diagram for SR-MnO_4_^−^ treatment system. Number 1 to 3 represents each chambers in the tank.

In the final part of this research, a contacting system was constructed to investigate the ability of SR-MnO_4_^−^ to effectively treat intermittent discharge water (Figure 6).

#### 3.5.1. Construction of Contact Tank

The specifically designed tank (45 cm × 30 cm × 30 cm) was rectangular and made of acrylic, which is a non-SDM absorbable material. The release of MnO_4_^−^ started in the first chamber when the SR-MnO_4_^−^ met water just 5 cm above the water surface. Baffles were used to divide the chambers. The first baffle attached to the first chamber forced water to pass only from the bottom of the tank to ensure that the reaction commenced within this chamber. The second chamber was the contact area, where mixing of the water allowed the accumulation and reaction with diffused MnO_4_^−^. Therefore, the second baffle height was adjusted depending on the time required for the MnO_4_^−^ to treat the SDM. The contact area was in the second and last chambers, which allowed precipitates to settle prior to wastewater release into the receiving watershed. 

#### 3.5.2. Remediation Experiment

Spiked-SDM in the discharge water was prepared to determine if the SR could treat multiple discharge events. We used a peristaltic pump model no. BT 100 2J (Baoding City, Hubei, China) connected with Masterflex Viton^®^ tubing (Coleparmer, IL, USA) to introduce water from an SDM solution reservoir at 50 mg L^−1^. Sampling was collected in the three chambers and at the end of the ditch to determine the overall removal efficiency of SDM in the effluent during each cycle. This allowed us to quantify the effect of residence times under dynamic conditions. Only the treatment of SRB+TKPP is selectively presented, along with the addition of MnO_4_^−^ solution in the matrices of either SDM-spiked DI water or wastewater. We imitated the use of SRB+TKPP by placing it in the holder so that the release of MnO_4_^−^ would only occur when needed (Figure 6).

## 4. Conclusions

In this study, we developed a slow-release oxidant consisting of permanganate (MnO_4_^−^), biodegradable wax, and a phosphate-based dispersing agent to degrade aquaculture antibiotics (SDM, OMP, and TMP). The details of our findings are provided in the following:

- The second-order degradation rates for these antibiotics were 0.128 s^−1^ M^−1^ for SDM, 0.097 s^−1^ M^−1^ for OMP, and 0.056 s^−1^ M^−1^ for TMP, proving that the MnO_4_^−^ efficiency for a variety of antibiotics depends upon their molecular structure.
- Manganese dioxide (MnO_2_) formed during treatment and enhanced SDM degradation by promoting surface-coordinated oxidization, but it also acted like a low permeable rind that reduced MnO_4_^−^ release.
- Solution pH beyond neutral (pH > 4–6) and the presence of natural scavengers, such as organic constituents, slowed and sometimes halted oxidative degradation.
- While the oxidant composite was effective in treating SDM, the biodegradable wax component still required some synthetic paraffin in the mixture (>12%) to provide structural integrity. Among the several biowax and mixing ratios tested, 80% beeswax in the SR composite (SRB) produced the most consistent permanganate release patterns.
- Both dispersing agents (TKPP, SHMP) mixed in the composite produced delayed MnO_2_ rind formation. By increasing this addition to more than 2.5% (>0.02 g) per SR weight, the cylindrical shape was compromised. Within this upper limit as a suitable amount, the addition of TKPP (SRB+TKPP) provided the best releasing concentration (up to 20% greater release) in the beeswax formulation. The Siepmann-Peppas model provided the best fit of MnO_4_^−^ release rates over 60 d.
- Using SRB+TKPP in the contact tank receiving the SDM-contaminated discharge water removed 80% of the SDM over three flushing cycles. These results confirmed that our SRB+TKPP formulation could provide sustained release of MnO_4_^−^ and warrant the proposed oxidant composite as a low-cost treatment technology suitable for treating antibiotic-contaminated discharge water.

## Figures and Tables

**Table 1:** Release model parameters for selected types of SR (paraffin and beeswax) with different chemical additions (TKPP or SHMP).

Model	Siepman-Peppas				Higuchi; t < 60 d			Higuchi; t ≤ 8 d			Noyes-Whitney			Weibull			
Generalized Eq.	$R_{t}=\alpha t^{\beta }$				$R_{t}=k\sqrt{t}$			$R_{t}=k\sqrt{t}$			$-\ln(1-R_{t})=kt$			$\ln\left[-\ln(1-R_{t}/100)\right]=\ln\alpha +\beta \ln t$			
Graphs	Figure S5				Figure S6			Figure S7			Figure S8			Figure S9			
Parameters	α	β	R^2^	r^2^_adj_	k	R^2^	r^2^_adj_	k	R^2^	r^2^_adj_	k	R^2^	r^2^_adj_	α	β	R^2^	r^2^_adj_
SC0	186.9	0.216	0.815	0.736	65.75	0.113	N/A	163.1	0.932	0.898	0.0361	N/A	N/A	0.3555	0.4325	0.8661	0.809
ST1	195.7	0.189	0.801	0.716	56.64	N/A	N/A	144.8	0.894	0.841	0.0276	N/A	N/A	0.3119	0.4536	0.8476	0.782
ST2	214.2	0.170	0.822	0.746	57.24	N/A	N/A	151.6	0.907	0.861	0.0296	N/A	N/A	0.3816	0.4015	0.8557	0.794
SS1	183.7	0.212	0.797	0.710	63.24	0.055	N/A	161.4	0.93	0.895	0.0359	N/A	N/A	0.3335	0.5269	0.8673	0.810
SS2	171.3	0.221	0.826	0.751	62.30	0.194	N/A	142.3	0.926	0.889	0.0304	N/A	N/A	0.3067	0.4877	0.8740	0.820
RC0	199.7	0.212	0.824	0.749	55.66	N/A	N/A	140.9	0.87	0.805	−0.0265	N/A	N/A	0.3639	0.3899	0.8171	0.739
RT1	207.0	0.189	0.804	0.720	52.79	N/A	N/A	152.4	0.86	0.790	−0.0266	N/A	N/A	0.3548	0.3924	0.8432	0.776
RT2	222.6	0.174	0.820	0.743	52.68	N/A	N/A	146.6	0.842	0.763	−0.0272	N/A	N/A	0.3243	0.4192	0.8786	0.827
RS1	196.4	0.209	0.797	0.710	53.27	N/A	N/A	135.8	0.929	0.894	−0.0243	N/A	N/A	0.3431	0.4012	0.8891	0.842
RS2	187.8	0.212	0.809	0.727	55.53	N/A	N/A	134.6	0.885	0.828	−0.0274	N/A	N/A	0.3713	0.3667	0.8923	0.846
BC0	137.4	0.313	0.933	0.904	73.47	0.791	0.739	97.40	0.984	0.976	−0.0396 (−0.0995)	0.371 (0.980)	0.214 (0.975)	0.8823	0.4831	0.9667	0.952
BT1	142.8	0.296	0.95	0.929	71.78	0.743	0.679	101.2	0.994	0.991	−0.0365 (−0.9901)	0.171 (0.978)	N/A (0.973)	0.2556	0.5198	0.9698	0.957
BT2	114.1	0.343	0.957	0.939	66.27	0.862	0.828	91.90	0.989	0.984	−0.0325 (−0.0727)	0.518 (0.952)	N/A (0.940)	0.2306	0.4737	0.9805	0.972
BS1	169.9	0.250	0.854	0.791	70.06	0.545	0.431	124.8	0.974	0.961	−0.0349 (−0.1162)	N/A (0.896)	N/A (0.870)	0.8908	0.4774	0.9281	0.897
BS2	216.3	0.189	0.85	0.786	64.85	0.119	N/A	153.5	0.966	0.949	−0.0355 (−0.1306)	N/A (0.631)	N/A (0.539)	0.4012	0.4398	0.8958	0.851
PC0	238.6	0.199	0.944	0.920	74.47	0.238	0.048	154.6	0.939	0.909	−0.0563 (−0.1603)	N/A (0.829)	N/A (0.786)	0.5897	0.3955	0.9732	0.962
PT1	241.2	0.186	0.934	0.906	70.18	0.083	N/A	166.7	0.935	0.903	−0.0465 (−0.1472)	N/A (0.480)	N/A (0.350)	0.5432	0.3993	0.9431	0.919
PT2	247.3	0.185	0.93	0.900	71.45	0.069	N/A	168.3	0.942	0.913	−0.0499 (−0.1747)	N/A (0.786)	N/A (0.733)	0.5666	0.4048	0.9291	0.899
PS1	234.5	0.201	0.933	0.904	73.64	0.229	0.036	160.0	0.961	0.942	−0.0509 (−0.1746)	N/A (0.859)	N/A (0.824)	0.5650	0.4044	0.9311	0.902
PS2	237.9	0.189	0.937	0.910	70.38	0.118	N/A	161.8	0.943	0.915	−0.0470 (−0.1594)	N/A (0.764)	N/A (0.705)	0.5638	0.3809	0.9382	0.912

## Data Availability

The authors confirm that the data supporting the findings of this study are available within the article.

## Supplementary Materials

The following supporting information can be downloaded at: https://www.mdpi.com/article/10.3390/antibiotics12061025/s1, Figure S1: Temporal changes in antibiotic relative concentrations (A; SDM 161.12 μM, B; OMP 36.45 μM, C; TMP 34.44 μM) following treatment with varying MnO_4_^−^ concentrations (0.315 to 5.033 mM for SDM or 0.189 to 27.181 mM for OMP or TMP) and loss of initial concentrations of antibiotics (D; SDM 16.11 to 161.12 μM, E; OMP 4.56 to 72.91 μM, F; TMP 4.56 to 72.91 μM) when treated with MnO_4_^−^ at 1.133 mM.; Figure S2: Observed kinetic rate constant (k_obs_) of each antibiotic degradation (A; SDM, B; OMP, or C; TMP) with presence of different synergetic antibiotics (as individual, SDM+OMP, or SDM+TMP) following treatment with MnO_4_^−^ at 180 mg L^−1^; Figure S3: Permanganate release concentration [Mixing ratio: Set A]; (A) Weight composition of SR-MnO_4_^−^ per batch (3.3 g) at different natural wax percentages. (B–E) Permanganate concentrations of each type of SR-MnO_4_^−^ at different natural wax percentages (20–100%) and at different timelines (0.25, 7, 28, and 56 d). Graphs (B–E) represent different types of natural wax in the mixture: (B) synthetic paraffin, (C) soy wax, (D) rice bran wax, and (E) beeswax; Figure S4: Permanganate concentrations of each type of SR-MnO_4_^−^ with different formulations of natural wax, synthetic paraffin, and chemical addition (TKPP or SHMP) for different timelines: (A) 0.25 d, (B) 15 d, and (C) 56 d. (D) Temporal changes of MnO_4_^−^ releasing concentration from selected types of SR; Figure S5: X-ray diffraction analysis of different types of SRB before and after soaking in SDM solution for 7 d; Figure S6: Temporal changes in MnO_4_^−^ concentration from each type of SR-MnO_4_^−^: (A) soy wax; (B) rice bran wax (C) beeswax; and (D) paraffin; Figure S7: Release pattern of MnO_4_^−^ concentration of each type of SR-MnO_4_^−^ plotted for Higuchi releasing model with all selected data (t < 60 d): (A) soy wax; (B) rice bran wax (C) beeswax; and (D) paraffin. Hatched boxes represented range of time that data may be fitted in linear regression; Figure S8: Linear regression of each type of SR-MnO_4_^−^ using Higuchi releasing model with selected data from t < 8 d: (A) soy wax; (B) rice bran wax (C) beeswax; and (D) paraffin; Figure S9: Release pattern of MnO_4_^−^ concentration of each type of SR-MnO_4_^−^ plotted for Noyes-Whitney releasing model: (A) soy wax; (B) rice bran wax (C) beeswax; and (D) paraffin. Hatched boxes represent range of time that data may be fitted in linear regression; Figure S10: Linear regression of each type of slow-release permanganate using Weibul releasing model: (A) soy wax; (B) rice bran wax (C) beeswax; and (D) paraffin; Table S1: Physiochemical characteristics of antibiotics; Table S2: Properties of TKPP and SHMP (Chokejaroenrat et al., 2014); Table S3: Physicochemical properties of aquaculture discharge wastewater; Table S4: Weight composition of SR-MnO_4_^−^ with chemical addition (TKPP or SHMP) per SR (0.75 g) [Mixing ratio: Set B]. References [51,52,53,54,55] are cited in the Supplementary Materials.
