# Supplementary material for: Developing a Slow-Release Permanganate Composite for Degrading Aquaculture Antibiotics
Source: Antibiotics (Basel). 2023 Jun 7;12(6):1025. doi: 10.3390/antibiotics12061025 (PMC10295586; doi:10.3390/antibiotics12061025)
Supplement: Supplementary file 1 [file antibiotics-12-01025-s001.zip › antibiotics-2423963-supplementary.pdf]

antibiotics

Supplementary materials

Developing a slow-release permanganate composite for degrading aquaculture antibiotics

Chainarong Sakulthaew [1], Chanat Chokejaroenrat [2,*], Sidaporn Panya [2], Apisit Songsasen [3], Kitipong Poomipuen [1], Saksit Imman [4], Nopparat Suriyachai [4], Torpong Kreetachat [4] and Steve Comfort [5]

10 Figures

4 Tables

- 15 pages –

antibiotics

MDPI

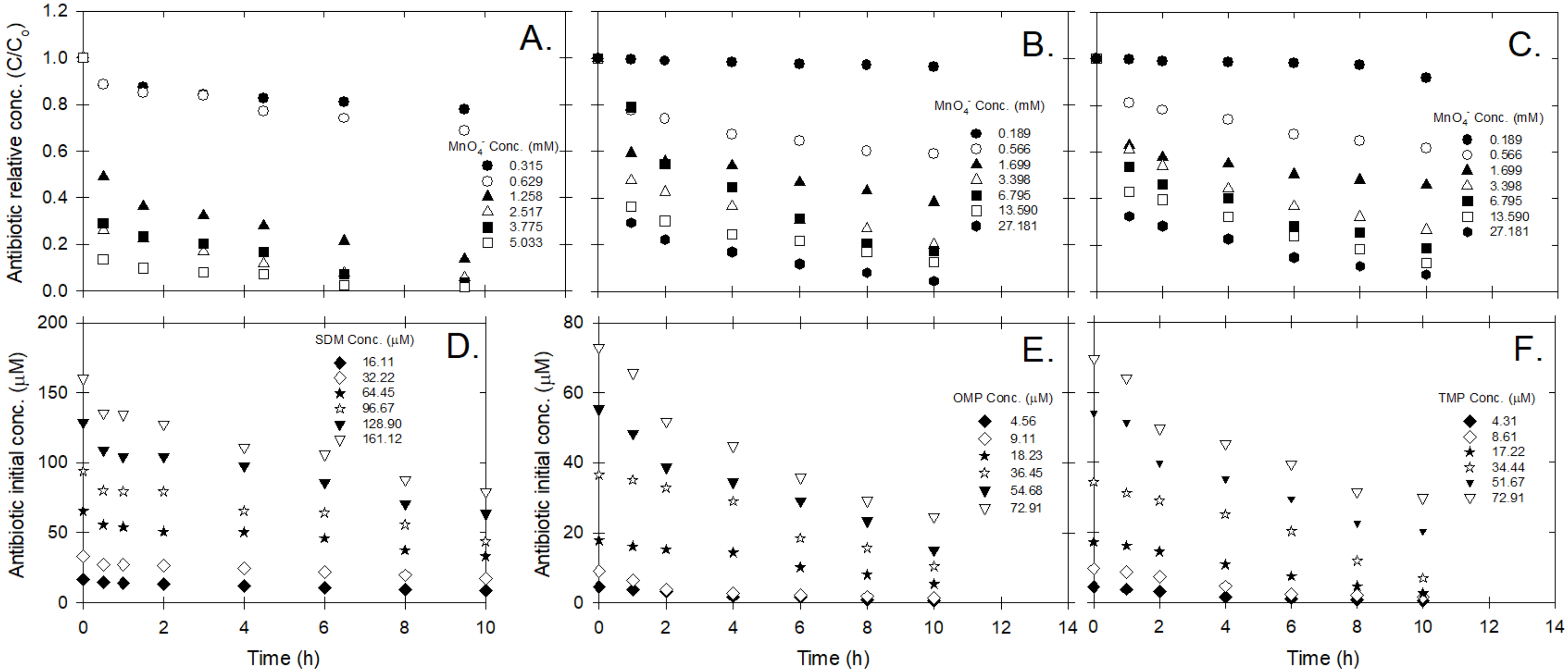

Figure S1. Temporal changes in antibiotic relative concentrations (**A**; Sulfadimethoxine, SDM 161.12 μM, **B**; Ormetoprim, OMP 36.45 μM, **C**; Trimethoprim, TMP 34.44 μM) following treatment with varying MnO_4^- concentrations (0.315 to 5.033 mM for SDM or 0.189 to 27.181 mM for OMP or TMP) and loss of initial concentrations of antibiotics (**D**; SDM 16.11 to 161.12 μM, **E**; OMP 4.56 to 72.91 μM, **F**; TMP 4.56 to 72.91 μM) when treated with MnO_4^- at 1.133 mM.

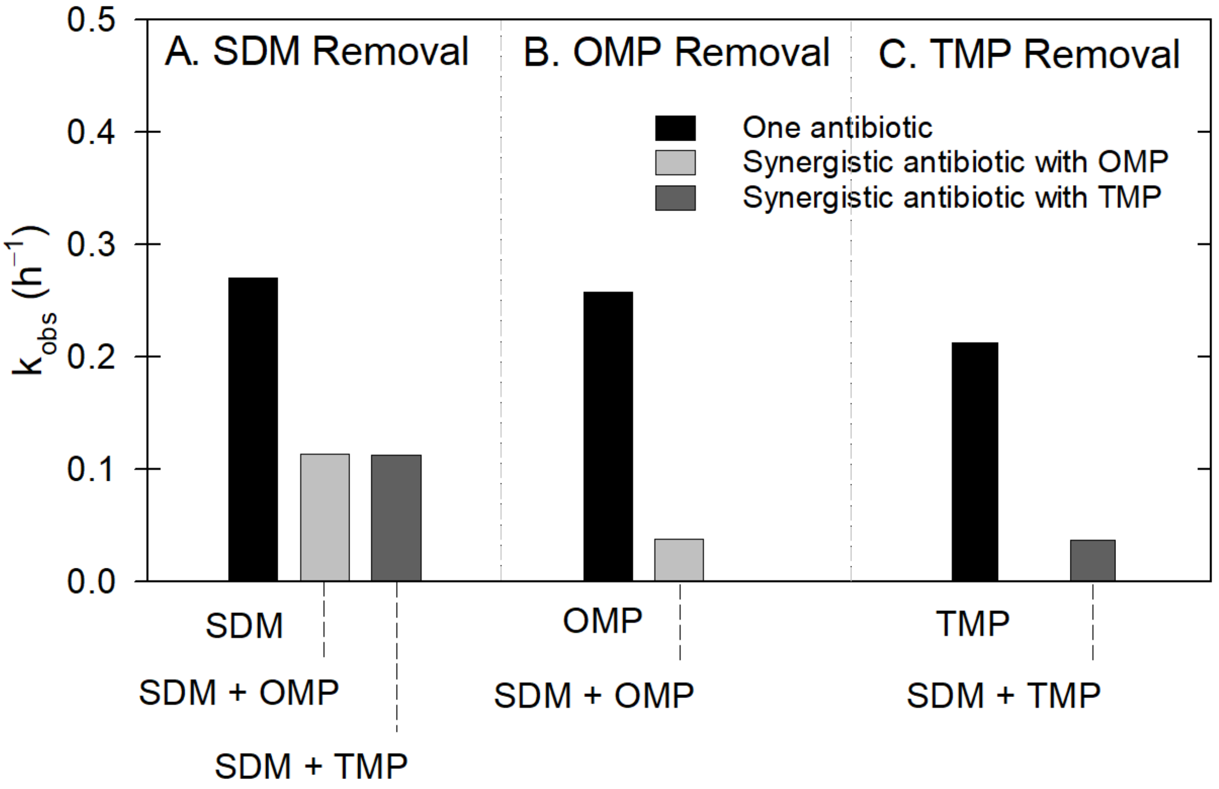

Figure S2. Observed kinetic rate constant (k_{obs}) of each antibiotic degradation (A; Sulfadimethoxine, SDM, B; Ormetoprim, OMP, or C; Trimethoprim, TMP) with presence of different synergetic antibiotics (as individual, SDM+OMP, or SDM+TMP) following treatment with MnO_4^- at 180 mg L^{-1}.

A

Natural wax percentage (%)	Weight ratio (per batch)			Total weight per batch (g)
	$KMnO_4$	Biowax	Paraffin	
20	2.3	0.2	0.8	3.3
40	2.3	0.4	0.6	3.3
60	2.3	0.6	0.4	3.3
80	2.3	0.8	0.2	3.3
100	2.3	1.0	0.0	3.3

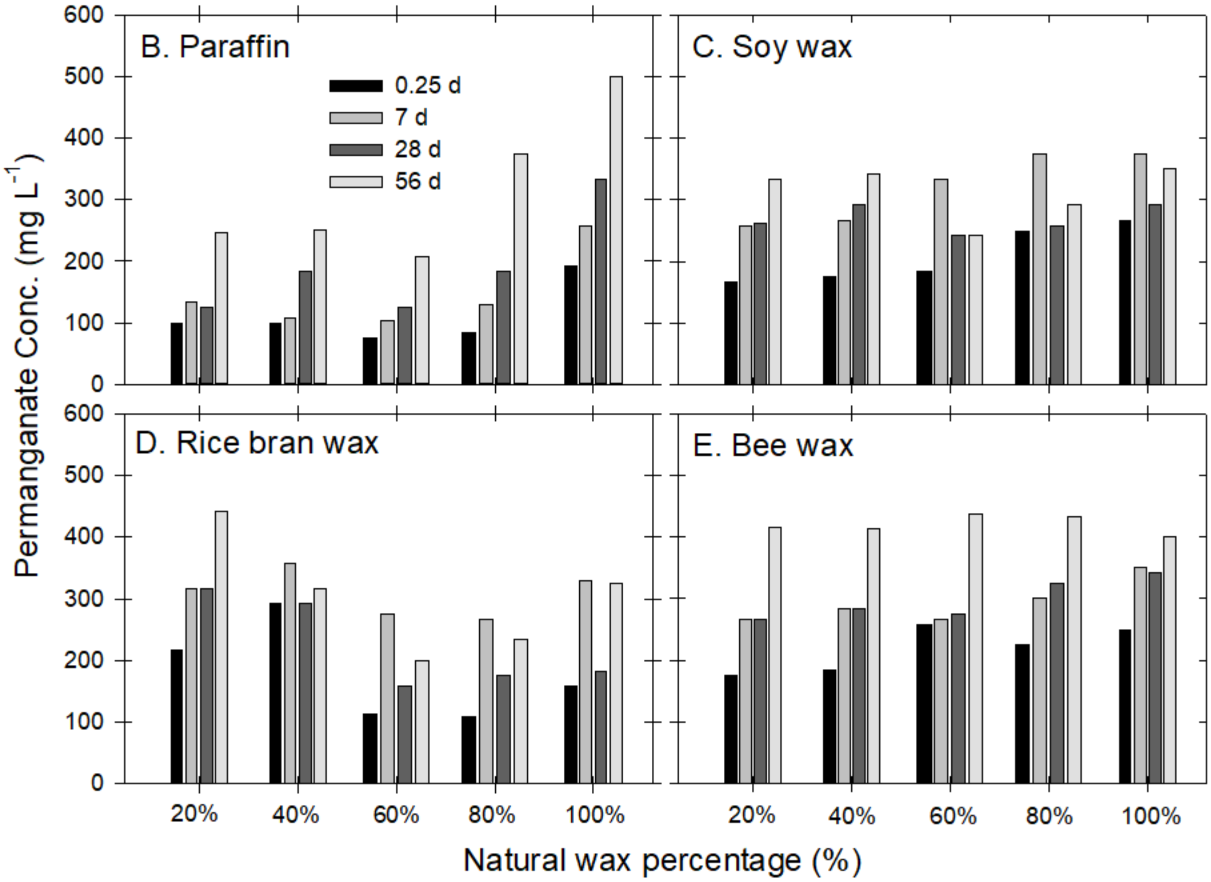

Figure S3. Permanganate release concentration [Mixing ratio: Set A]; **(A)** Weight composition of SR-MnO_4^- per batch (3.3 g) at different natural wax percentages. (B–E) Permanganate concentrations of each type of SR-MnO_4^- at different natural wax percentages (20–100%) and at different timelines (0.25, 7, 28, and 56d). Graphs **(B–E)** represent different types of natural wax in the mixture: **(B)** synthetic paraffin, **(C)** soy wax, **(D)** rice bran wax, and **(E)** beeswax.

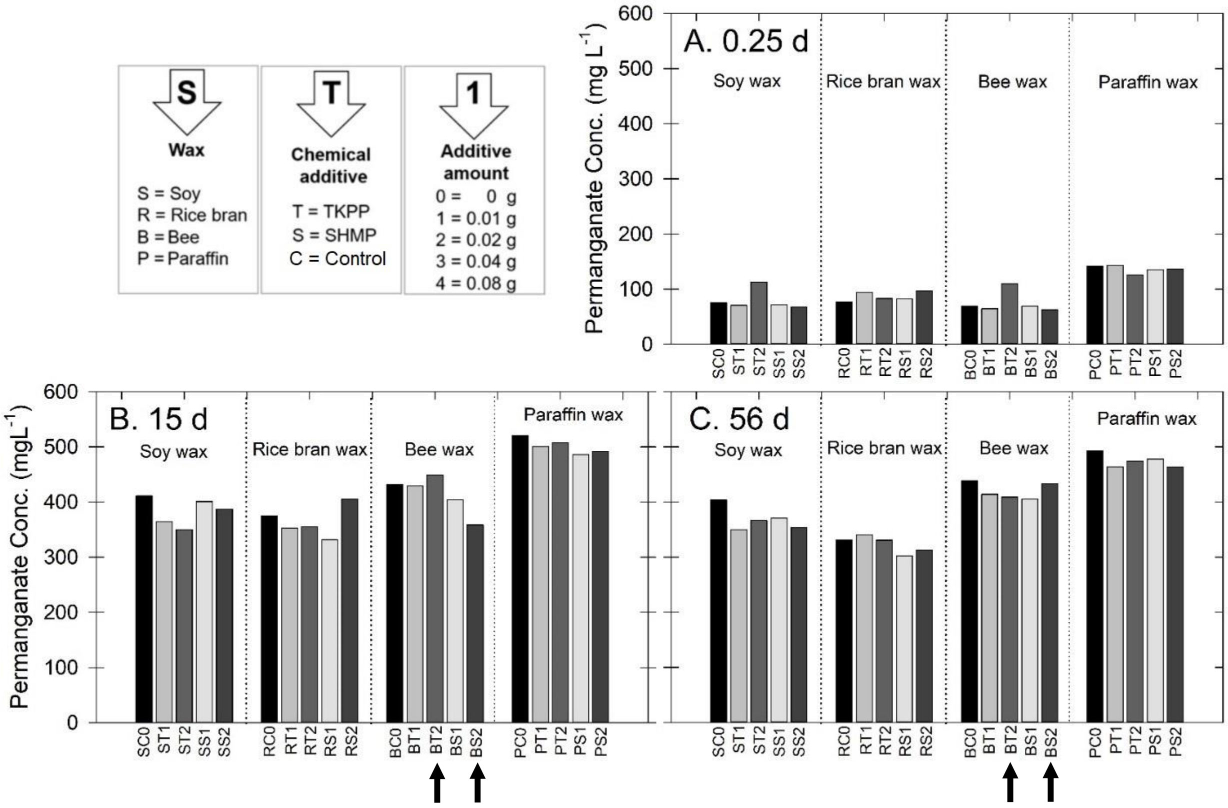

Figure S4. Permanganate concentrations of each type of SR-MnO_4^- with different formulations of natural wax, synthetic paraffin, and chemical addition (TKPP or SHMP) for different timelines: **(A)** 0.25 day, **(B)** 15 day, and **(C)** 56 day.

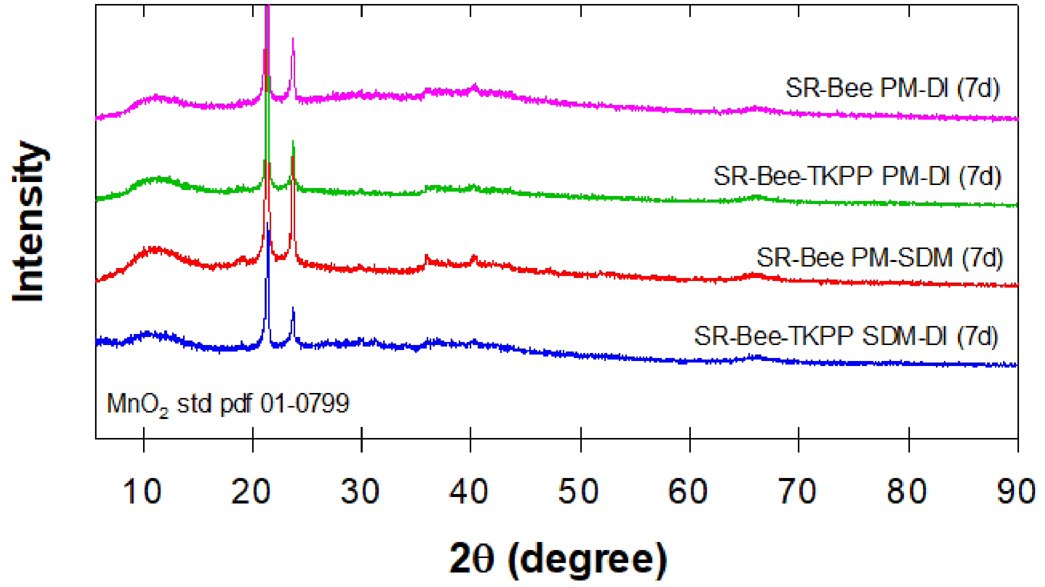

Figure S5. X-ray diffraction analysis of different types of slow-release beewax (SRB) before and after soaking in SDM solution for 7 d. (Bee: beewax, PM: permanganate, TKPP: Tetrapotassium pyrophosphate, SDM: Sulfadimethoxine, DI: distilled water)

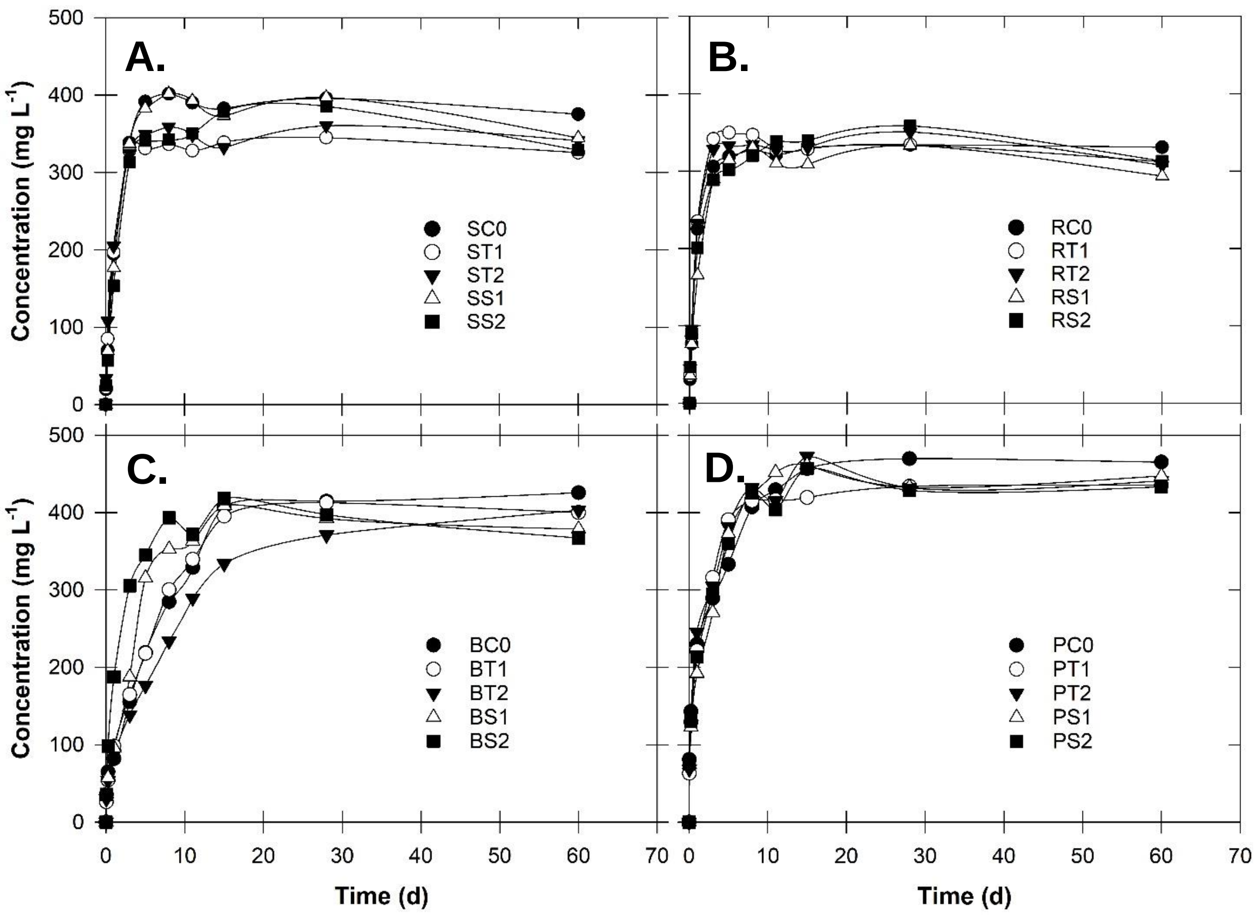

Figure S6. Temporal changes in MnO_4^- concentration from each type of SR-MnO_4^-: **(A)** soy wax; **(B)** rice bran wax **(C)** beeswax; and **(D)** paraffin. The explanation of slow-release sample abbreviation (e.g., SC0, ST1, etc.) is provided in the Figure S4.

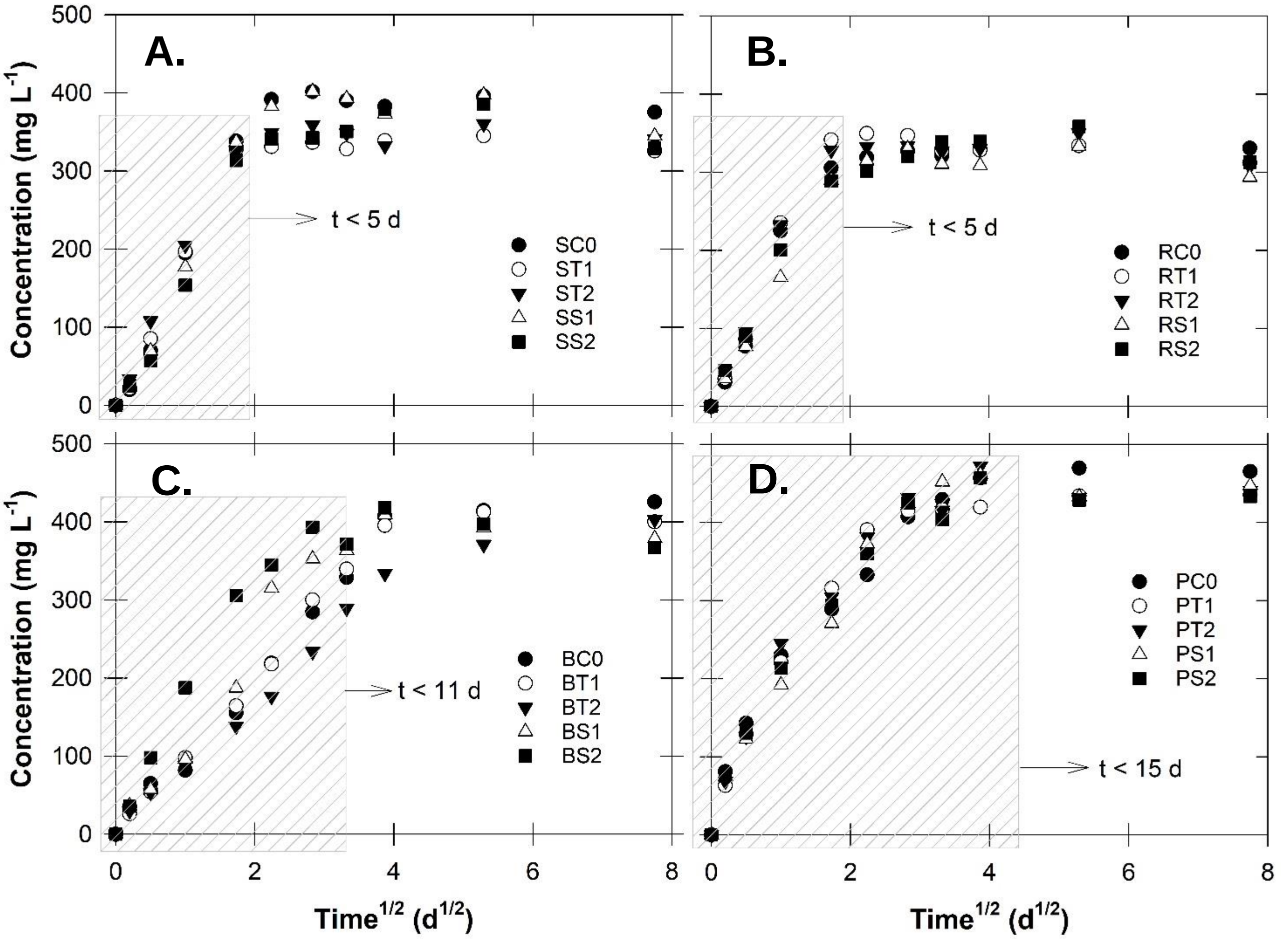

Figure S7. Release pattern of MnO_4^- concentration of each type of SR-MnO_4^- plotted for Higuchi releasing model with all selected data (t < 60 d): **(A)** soy wax; **(B)** rice bran wax **(C)** beeswax; and **(D)** paraffin. Hatched boxes represented range of time that data may be fitted in linear regression. The explanation of slow-release sample abbreviation (e.g., SC0, ST1, etc.) is provided in the Figure S4.

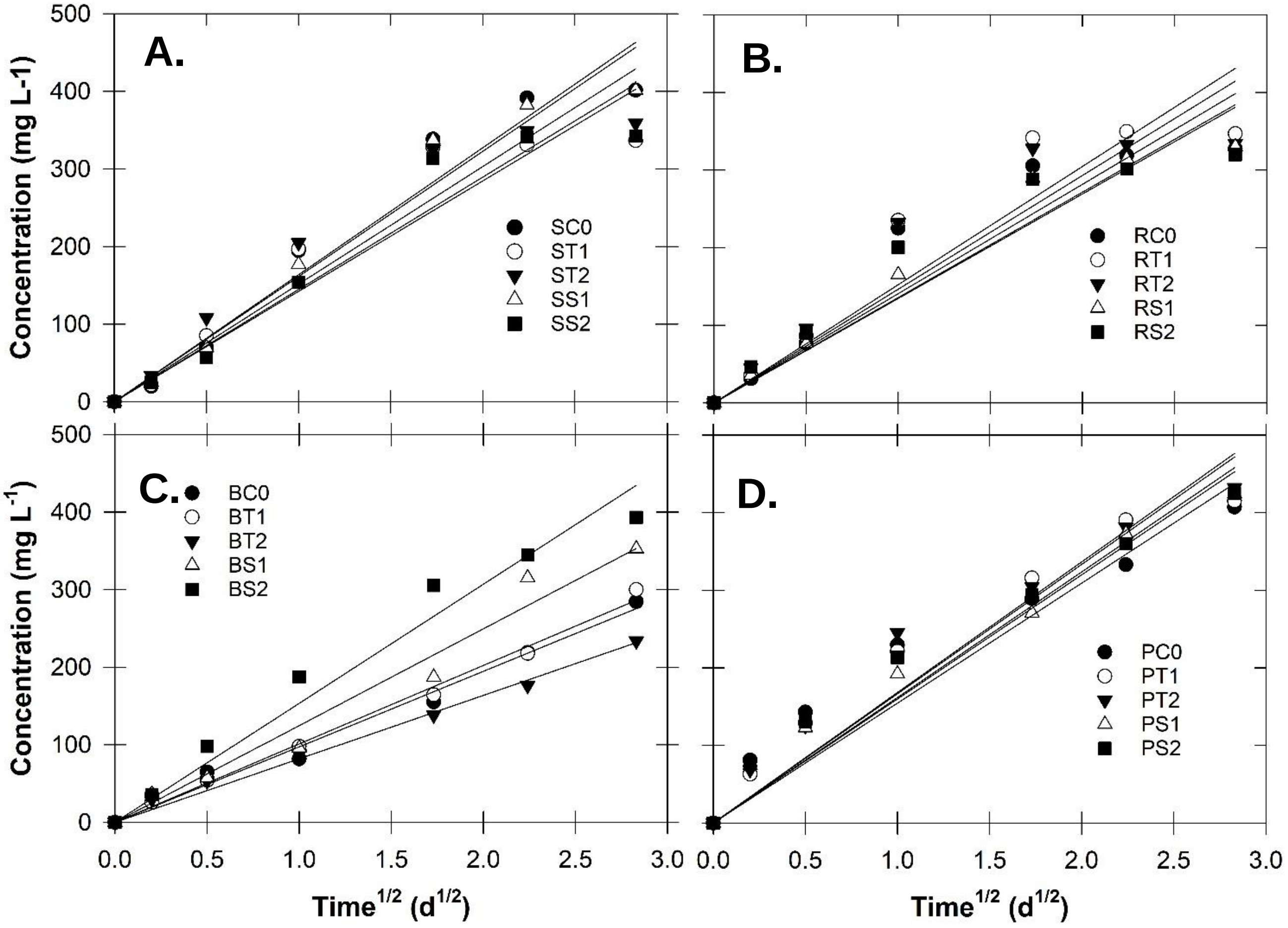

Figure S8. Linear regression of each type of SR-MnO_4^- using Higuchi releasing model with selected data from t < 8 d: **(A)** soy wax; **(B)** rice bran wax **(C)** beeswax; and **(D)** paraffin. The explanation of slow-release sample abbreviation (e.g., SC0, ST1, etc.) is provided in the Figure S4.

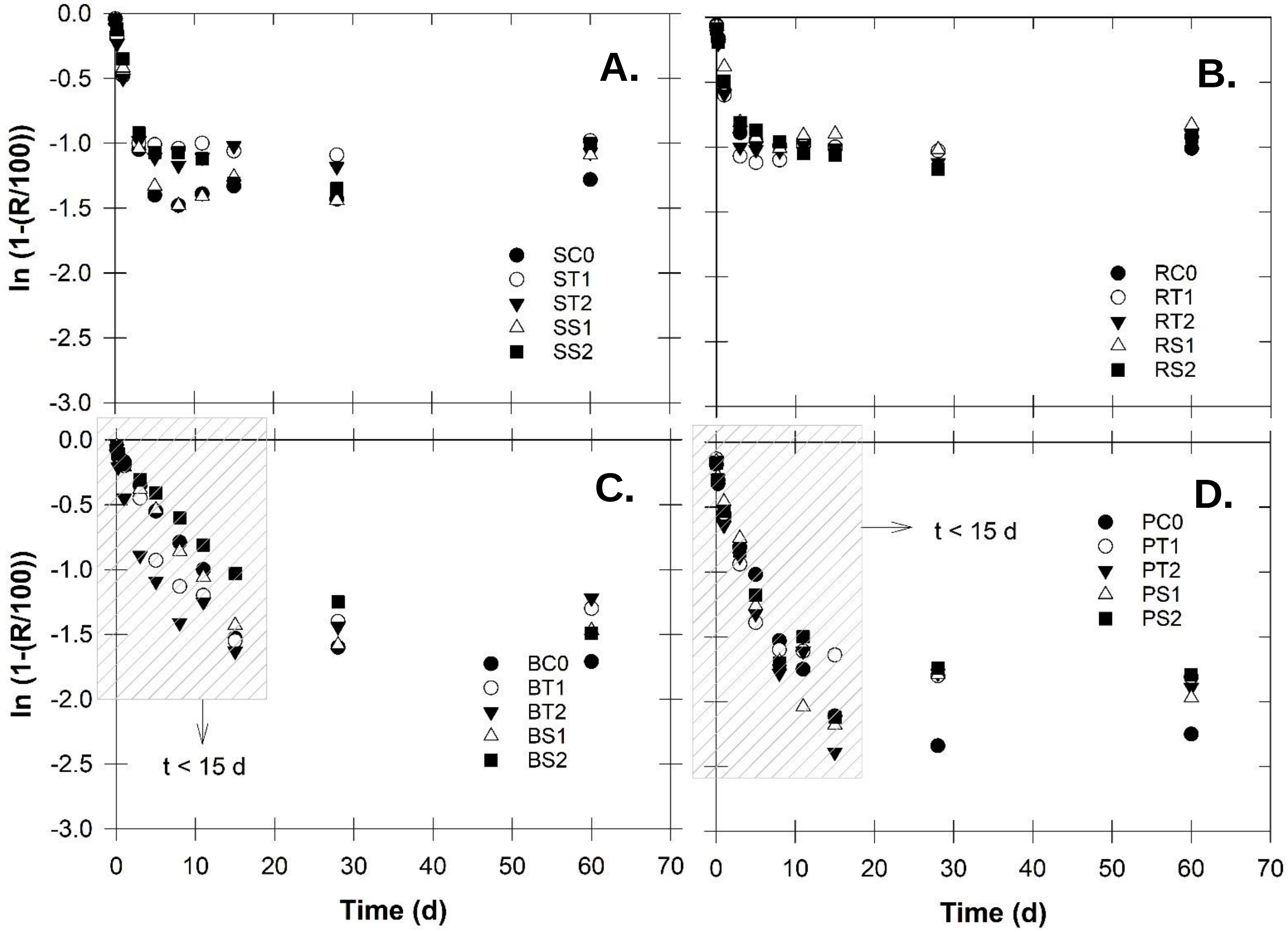

Figure S9. Release pattern of MnO_4^- concentration of each type of SR-MnO_4^- plotted for Noyes-Whitney releasing model: **(A)** soy wax; **(B)** rice bran wax **(C)** beeswax; and **(D)** paraffin. Hatched boxes represent range of time that data may be fitted in linear regression. The explanation of slow-release sample abbreviation (e.g., SC0, ST1, etc.) is provided in the Figure S4.

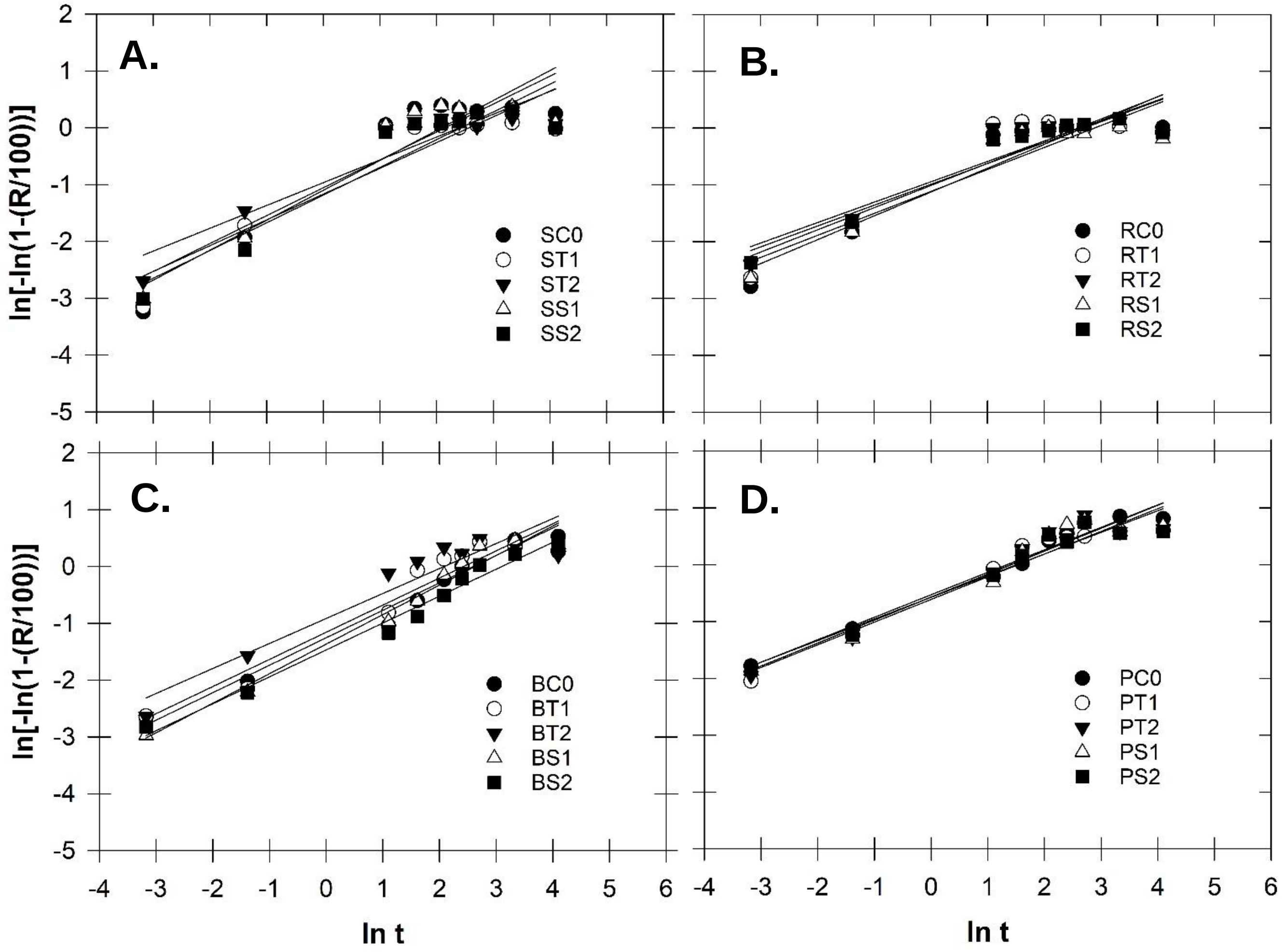

Figure S10. Linear regression of each type of slow-release permanganate using Weibul releasing model: **(A)** soy wax; **(B)** rice bran wax **(C)** beeswax; and **(D)** paraffin. The explanation of slow-release sample abbreviation (e.g., SC0, ST1, etc.) is provided in the Figure S4.

Table S1. Physiochemical characteristics of antibiotics.

Antimicrobial; Abbreviation (CAS Number)	Chemical Structure	Molecular Formula	MW (g/mol)	Water Solubility at 25°C (mg/L)	Henry's Constant (atm-m^{3}·mol^{-1})	Log K_{ow}	pK_{a1} (pK_{a2})	Wavelength (nm)
Sulfadimethoxine;SDM[a] (122-11-2)	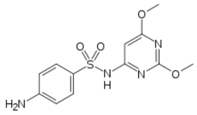	$C_{12}H_{14}N_4O_4S$	310.33	343	1.30×10^{-14}	1.63	2.4 (6.0)	270[e]
Ormetoprim; OMP[a] (6981-18-6)	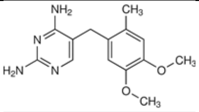	$C_{14}H_{18}N_4O_2$	274.32	1,540	4.45×10^{-13}	1.23	7	270[e]
Trimethoprim; TMP[b] (738-70-5)	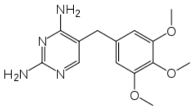	$C_{14}H_{18}N_4O_3$	290.32	400	1.32×10^{-6} (Vapor pressure)[c]	0.91	3.2 (6.7)[d]	230[e]

References: [a]Sanders et al. [1]; [b]Straub, [2]; [c]Gros et al. [3]; [d]Qiang and Adams [4]; [e]Samuelsen et al. [5]

Table S2. Properties of TKPP and SHMP (Chokejaroenrat et al. [6])

Chemical	Molecular Structure	Molecular Formula	Description	M.W. (g mol^{-1})	Density (g cm^{-3})	Solubility (mg L^{-1})	Manufacturer
Sodium hexameta-phosphate(SHMP)	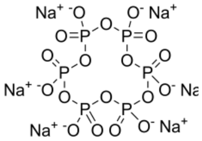	$(NaPO_3)_6$	Dispersing agent	611.77	2.484	Soluble	Sigma Aldrich
Tetrapotassium pyrophosphate (TKPP)	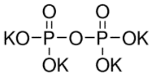	$K_4P_2O_7$	Dispersing agent	330.34	-	Highly soluble	Carus Corporation

Table S3. Physicochemical properties of aquaculture discharge wastewater.

Parameter	Unit	Value
pH	-	7.91
Turbidity	NTU	84
Conductivity	$\mu S\ cm^{-1}$	141
Total kjeldahl nitrogen (TKN)	mgL^{-1}	7.68
Nitrate	mgL^{-1}	0.1
Sulfate	mgL^{-1}	6.03
Chloride	mgL^{-1}	110.83
Phosphate	$mg\text{-}P\ L^{-1}$	7.45
Salinity	PSU	0.7
Total phosphorus	mgL^{-1}	0.46
Ammonia-nitrogen (mg/l)	mgL^{-1}	0.89
Dissolved oxygen	mgL^{-1}	8.8
Total alkalinity	mgL^{-1} as $CaCO_3$	31.6
Total suspended solid	mgL^{-1}	282
Total dissolve solid (TDS)	mgL^{-1}	147
Total organic carbon (TOC)	$mg\text{-}C\ L^{-1}$	62.4
Biological oxygen demand (BOD)	mgL^{-1}	22.6
Chemical oxygen demand (COD)	mgL^{-1}	225

Table S4. Weight composition of SR-MnO_4^- with chemical addition (Tetrapotassium pyrophosphate, TKPP, or Sodium hexametaphosphate, SHMP) per SR (0.75 g) [Mixing ratio: Set B].

SR-MnO_4^-	Weight ratio (per slow-release) (g)				
	$KMnO_4$	Biowax	Paraffin	TKPP	SHMP
XC0	0.525 (70.0%)	0.135 (18.0%)	0.090 (12.0%)	-	-
XT1	0.525 (70.0%)	0.125 (16.7%)	0.090 (12.0%)	0.010 (0.0%)	-
XT2	0.525 (70.0%)	0.115 (15.3%)	0.090 (12.0%)	0.020 (1.3%)	-
XT3	0.525 (70.0%)	0.095 (12.7%)	0.090 (12.0%)	0.040 (2.7%)	-
XT4	0.525 (70.0%)	0.055 (7.3%)	0.090 (12.0%)	0.080 (5.3%)	-
XS1	0.525 (70.0%)	0.135 (18.0%)	0.090 (12.0%)	-	0.010 (0.0%)
XS2	0.525 (70.0%)	0.125 (16.7%)	0.090 (12.0%)	-	0.020 (1.3%)
XS3	0.525 (70.0%)	0.115 (15.3%)	0.090 (12.0%)	-	0.040 (2.7%)
XS4	0.525 (70.0%)	0.095 (12.7%)	0.090 (12.0%)	-	0.080 (5.3%)

Remark

1. First letter of each slow-release PM (X) represents the type of wax (P = paraffin; S = soy wax; R = rice bran, wax; B = Beeswax)
2. Percentages in parentheses were calculated based on amount of component in one slow-release PM (0.75 g).

Reference

[1] Sanders, S.; Srivastava, P.; Feng, Y.; Dane, J.; Basile, J.; Barnett, M. Sorption of the Veterinary Antimicrobials Sulfadimethoxine and Ormetoprim in Soil. *Journal of environmental quality* **2008**, *37*, 1510-1518, doi:10.2134/jeq2007.0215.

[2] Straub, J.O. An Environmental Risk Assessment for Human-Use Trimethoprim in European Surface Waters. *Antibiotics (Basel)* **2013**, *2*, 115-162, doi:10.3390/antibiotics2010115.

[3] Gros, M.; Petrović, M.; Barceló, D. Development of a multi-residue analytical methodology based on liquid chromatography-tandem mass spectrometry (LC-MS/MS) for screening and trace level determination of pharmaceuticals in surface and wastewaters. *Talanta* **2006**, *70*, 678-690, doi:10.1016/j.talanta.2006.05.024.

[4] Qiang, Z.; Adams, C. Potentiometric determination of acid dissociation constants (pKa) for human and veterinary antibiotics. *Water Research* **2004**, *38*, 2874-2890, doi:https://doi.org/10.1016/j.watres.2004.03.017.

[5] Samuelsen, O.B.; Lunestad, B.T.; Ervik, A.; Fjelde, S. Stability of antibacterial agents in an artificial marine aquaculture sediment studied under laboratory conditions. *Aquaculture* **1994**, *126*, 283-290, doi:https://doi.org/10.1016/0044-8486(94)90044-2

[6] Chokejaroenrat, C.; Comfort, S.; Sakulthaew, C.; Dvorak, B. Improving the treatment of non-aqueous phase TCE in low permeability zones with permanganate. *J. Hazard. Mater.* **2014**, *268*, 177–184, https://doi.org/10.1016/j.jhazmat.2014.01.007.
